# Supplementary material for: Materializing rival ground states in the barlowite family of kagome magnets: quantum spin liquid, spin ordered, and valence bond crystal states
Source: NPJ Quantum Mater. Author manuscript; Available in PMC 2020 Oct 15. (PMC7558222)
Supplement: Supp1 [file NIHMS1616319-supplement-Supp1.pdf]

Supplementary Material for: Materializing Rival Ground States in the Barlowite Family of Kagome Magnets: Quantum Spin Liquid, Spin Ordered, and Valence Bond Crystal States

Rebecca W. Smaha,[1,2] Wei He,[1,3] Jack Mingde Jiang,[1,4] Jiajia Wen,[1]
Yi-Fan Jiang,[1] John P. Sheckelton,[1] Charles J. Titus,[5] Suyin Grass Wang,[6]
Yu-Sheng Chen,[6] Simon J. Teat,[7] Adam A. Aczel,[8,9] Yang Zhao,[10,11]
Guangyong Xu,[10] Jeffrey W. Lynn,[10] Hong-Chen Jiang,[1] and Young S. Lee[1,4]

[1]*Stanford Institute for Materials and Energy Sciences, SLAC National Accelerator Laboratory, Menlo Park, California 94025, USA*
[2]*Department of Chemistry, Stanford University, Stanford, California 94305, USA*
[3]*Department of Materials Science and Engineering, Stanford University, Stanford, California 94305, USA*
[4]*Department of Applied Physics, Stanford University, Stanford, California 94305, USA*
[5]*Department of Physics, Stanford University, Stanford, California 94305, USA*
[6]*NSF's ChemMatCARS, Center for Advanced Radiation Sources, c/o Advanced Photon Source/ANL, The University of Chicago, Argonne, Illinois 60439, USA*
[7]*Advanced Light Source, Lawrence Berkeley National Laboratory, Berkeley, California 94720, USA*
[8]*Neutron Scattering Division, Oak Ridge National Laboratory, Oak Ridge, Tennessee 37831, USA*
[9]*Department of Physics and Astronomy, University of Tennessee, Knoxville, Tennessee 37996, USA*
[10]*NIST Center for Neutron Research, National Institute of Standards and Technology, Gaithersburg, MD 20899-6102 USA*
[11]*Department of Materials Science and Engineering, University of Maryland, College Park, Maryland 20742, USA*

CONTENTS

I. Composition and Structural Overview 3
A. Barlowite **1** 3
B. Barlowite **2** 4
C. Zn-substituted barlowite 4

II. Synchrotron Single Crystal X-ray Diffraction 6
A. Barlowite **2** 6
B. Zn-substituted barlowite 6

III. Powder X-ray and Neutron Diffraction of Barlowite 14

IV. Powder X-ray and Neutron Diffraction of Zn-Substituted Barlowite 23

V. Physical Properties of Barlowite and Zn-Substituted Barlowite 28
A. Magnetic measurements of deuterated barlowite **2** 36

VI. Elastic Neutron Scattering of Barlowite **2** 37
A. Background subtraction and fitting methods for the superlattice peak 37
B. Peak broadening of superlattice nuclear Bragg peaks and magnetic Bragg peaks 37
C. Resolution function calculation and magnetic structure refinement method 38
D. Detailed refinement results of the magnetic models 41
E. The orthorhombic model 49
F. Including interlayer moments into calculations for barlowite **2** 52

VII. First-order phase transition between the pinwheel VBC and QSL 54

References 55

I. COMPOSITION AND STRUCTURAL OVERVIEW

Barlowite and Zn-substituted barlowite ($Cu_3Zn_xCu_{1-x}(OH)_6FBr$) were synthesized as previously described.[33] Samples synthesized using $Cu_2(OH)_2CO_3$ are referred to as **1** and $\mathbf{Zn}_{0.95}$ (powder and small crystals) for barlowite and Zn-substituted barlowite, respectively. Crystals grown using CuF_2 are referred to as **2** for barlowite and $\mathbf{Zn}_{0.56}$ for Zn-substituted barlowite. The subscript reflects the amount of Zn substitution as measured by inductively coupled plasma atomic emission spectroscopy (ICP-AES). The deuterated powder sample (denoted $\mathbf{Zn}_{0.95}$, $x = 0.95$) has nearly a full equivalent of Zn, which is comparable to (but higher than) that reported in herbertsmithite.[27] Our current synthesis of large single crystals yields lower Zn substitution levels: $x = 0.56$, denoted $\mathbf{Zn}_{0.56}$ ($Cu_{3.44}Zn_{0.56}(OH)_6FBr$). A summary of all samples studied in this work as well as herbertsmithite and its all-Cu parent, clinoatacamite, is presented in Table 1.

A. Barlowite 1

To resolve open questions in the literature regarding the low-temperature structure of barlowite, we have undertaken a comprehensive investigation of barlowite **1**. Previous neutron powder diffraction (NPD) reports assign it to orthorhombic space group *Pnma* (No. 62)[34, 35, 41], but others conclude that space group *Cmcm* (No. 63) is plausible.[40] We employ a combination of high resolution synchrotron powder and single crystal X-ray diffraction (PXRD and SCXRD) as well as neutron powder diffraction (NPD) to definitively determine that our sample of barlowite **1** has a reversible phase transition at $T \approx 265$ K to *Pnma.* Figure 1b in the main text shows the emergence in synchrotron PXRD of superlattice peaks and orthorhombic peak splitting related to this transition, which is confirmed by low-temperature synchrotron SCXRD and Rietveld co-refinements of PXRD and NPD data (see Figures 2–4; Tables 2–3, 8).

For several samples similar to barlowite **1** but synthesized using different reagents and temperature profile—which can affect the magnetic properties[33]—the recently proposed low-temperature *Pnma* orthorhombic structures based on NPD contain only one (off center) interlayer Cu^{2+} site, which is fully occupied.[34, 35] However, by combining high-resolution synchrotron SCXRD data with co-refined PXRD and NPD data, we find a different model of the interlayer Cu^{2+}. We determine that in barlowite **1** the interlayer Cu^{2+}s become disordered over three symmetry inequivalent sites with unequal occupancies (Figure 1a in the main text). The much higher resolution of synchrotron SCXRD and PXRD compared to NPD lends credence to the accuracy of this model; using a model with only one interlayer site worsens the R_1 of our SCXRD model from 2.23% to 14.64%. The occupancies of these three disordered interlayer sites vary slightly in different measurements: they are (52%, 33%, 15%) from the $T = 15$ K SCXRD refinement and (83%, 11%, 5%) from the low-temperature ($T_{\mathrm{NPD}} = 2$ K and $T_{\mathrm{PXRD}} = 13$ K) co-refinement (Tables 3, 8). This difference can be ascribed to small variance in the crystals. The overarching trend, however, is that one site dominates the occupancy—likely why the previous NPD models only identify one interlayer site.

While we are in broad agreement with the literature as to the *Pnma* symmetry of orthorhombic barlowite,[34, 35, 41] it is unsurprising that there may be inconsistencies between structural models measured for different samples given the observed dependence of barlowite's physical

properties upon factors such as reactant stoichiometry and synthetic conditions.[33] The proposed *Cmcm* symmetry of a sample similar to barlowite **1** was determined using several local probe measurements at room temperature.[40] Our room temperature $P6_3/mmc$ model is consistent with the ^{19}F magic angle spinning (MAS) NMR data, and our previously published FTIR spectroscopy data is also consistent with the reported IR data.[33, 40] However, the ^{1}H MAS NMR and electron diffraction data are incompatible with $P6_3/mmc$.[40] Attempting to consolidate structural models for samples synthesized in distinct ways may not be valid. Local probe measurements such as solid-state ^{1}H MAS NMR would be required to ensure that each sample has true $P6_3/mmc$ symmetry.

B. Barlowite 2

All structural measurements support the lack of an orthorhombic transition in barlowite **2**; superlattice peaks related to hexagonal space group $P6_3/m$ are observed in SCXRD (Figure 1) and in neutron scattering (Figure 1d in the main text and Figure 20). No evidence of orthorhombic splitting is observed in the PXRD data, implying hexagonal symmetry is preserved (Section III). SCXRD and PXRD refinements on the same sample at different temperatures (Tables 3, 9) show slight variation in the relative occupancies of the interlayer sites, ranging from (43%, 33%, 24%) from SCXRD at $T = 15$ K to (42%, 31%, 27%) and (44%, 30%, and 27%) from PXRD at $T = 15$ and 99 K, respectively. These deviations are much smaller than those observed in barlowite **1** and further support the lower degree of symmetry lowering observed in this subtle hexagonal phase transition compared to barlowite **1**'s orthorhombic transition.

C. Zn-substituted barlowite

Both compositions of Zn-substituted barlowite ($\mathbf{Zn}_{0.95}$ and $\mathbf{Zn}_{0.56}$) crystallize in space group $P6_3/mmc$ at all temperatures measured. Slight variations in the site occupancies of the two interlayer sites (centered D_{3h} and the set of three off-center C_{2v} sites observed in all-Cu barlowite) occur in both samples. The following discussion assumes that the centered site is occupied by Zn^{2+} and the off-center sites are occupied by Cu^{2+}, consistent with their different Jahn-Teller activities.

SCXRD refinements on the same crystal of $\mathbf{Zn}_{0.56}$ at different temperatures show slight variation in the relative occupancies of the interlayer sites, ranging from (59% Zn, 41% Cu) at $T = 300$ K to (50% Zn, 50% Cu) at $T = 100$ K (Tables 2, 4). Rietveld refinements of PXRD data on the sample show similar deviations, ranging from (62% Zn, 38% Cu) at $T = 90$ K to (58% Zn, 42% Cu) at $T = 295$ K (Table 12). The average formula for $\mathbf{Zn}_{0.56}$ from all diffraction measurements is $Cu_{3.43}Zn_{0.57}(OH)_6FBr$, consistent with the ICP-AES results.

Rietveld co-refinements of NPD and synchrotron PXRD data of $\mathbf{Zn}_{0.95}$ were performed assuming full occupancy of Zn on the interlayer site. The single crystal measured in SCXRD has (85% Zn, 15% Cu) on the interlayer. Although this is reasonably close to the bulk ICP-AES results ($Cu_{3.05}Zn_{0.95}(OH)_6FBr$), this crystal may not be an accurate representation of the bulk (see Section II B).

TABLE 1: Comparison of selected parameters of barlowite, Zn-substituted barlowite, herbertsmitite, and clinoatacamite

**Compound**	Barlowite **1**	Barlowite **2**	**Zn**$_{0.56}$	**Zn**$_{0.95}$	Herbertsmithite	Clinoatacamite
**Formula**	$Cu_4(OD)_6FBr$	$Cu_4(OH)_6FBr$	$Cu_{3.44}Zn_{0.56}(OH)_6FBr$	$Cu_{3.05}Zn_{0.95}(OD)_6FBr$	$Cu_3Zn(OH)_6Cl_2$	$Cu_2(OD)_3Cl$
**Reference**	This work	This work	This work	This work	Ref. [15]	Ref. [61]
**Technique**	SCXRD	SCXRD	SCXRD	PXRD/NPD	SCXRD	NPD
**Temperature**	17 K	15 K	13 K / 2 K	100 K	130 K	2 K
**Crystal System**	Orthorhombic	Hexagonal	Hexagonal	Hexagonal	Trigonal	Monoclinic
**Space Group**	*Pnma*	$P6_3/m$	$P6_3/mmc$	$P6_3/mmc$	$R\bar{3}m$	$P2_1/n$
Kagome stacking	AA	AA	AA	AA	ABC (offset by 60°)	AB (offset by 60°)
No. of distinct kagome Cu's	2	2	1	1	1	1
No. of distinct kagome triangles	1	2	1	1	1	1
Are kagome triangles equilateral?	No	25% Yes, 75% No	Yes	Yes	Yes	No
Ratio of shortest to longest Cu–Cu bond length	1.0004	1 (25%), 1.0004 (75%)	1	1	1	1.0076
Average Cu–O–Cu bond angle around kagome triangle	117.24°	117.20° (25%), 117.24° (75%)	116.93°	117.01°	119.1°	119.64°
Cu–O–Cu bond angles around inequivalent kagome triangles	115.97(10)°, 117.30(11)°, 118.45(11)°	116.44(5)°, 117.20(5)°, 118.04(5)°	n/a	n/a	n/a	117.11(15)°, 117.42(12)°, 124.39(17)°
Out-of-plane buckling of oxygen[a]	27.48°	26.33° (25%), 26.29° (75%)	26.573°	26.53°	26.51°	28.24°

[a]Calculated as the dihedral angle between the kagome plane and the plane containing Cu1–O1–Cu1

II. SYNCHROTRON SINGLE CRYSTAL X-RAY DIFFRACTION

Low-temperature synchrotron X-ray diffraction measurements were performed on single crystals of barlowite **1**, barlowite **2**, and Zn-substituted barlowite $\mathbf{Zn}_{0.56}$ and $\mathbf{Zn}_{0.95}$ at $T = 14$ K or $T = 100$ K; $\mathbf{Zn}_{0.56}$ was also measured at $T = 300$ K (Table 2). Barlowite **1** and **2** were previously measured at $T = 300$ K; the details of these measurements and crystallographic information files (CIFs) can be found in Ref. [33]. Crystallographic information is tabulated in Table 3 for barlowite **1** and **2** and in Table 4 for Zn-substituted barlowite. Selected bond angles and distances are found in Tables 6–7.

A. Barlowite 2

Precession images for barlowite **2** at $T = 300$ K and $T = 15$ K are displayed in Figure 1. All expected superlattice peak positions arising from space group *P*6_3/*m* are circled in red; the presence of peaks at these positions indicates the presence of this low-temperature phase transition. We do observe a very weak [0 0 l] ($l \neq 2n$ peak in barlowite **2** at low temperature, although the reflection conditions for both *P*6_3/*m* and *P*6_3/*mmc* are $00l, l = 2n$. Their presence suggests that the stacking has subtle imperfections or that there may be very weak doubling of the *c*-axis: on average, the [0 0 -1] peak is 5500 times weaker than the allowed [0 0 ±2] peaks and 8000 times weaker than the [±1 0 0] peaks. Of the two equivalent reflections, one has an intensity within error of 0 (0.01(1)) and the other's is very close (0.04(2)).

B. Zn-substituted barlowite

We report here the first crystal structure of single crystalline Zn-substituted barlowite with no magnetic order down to $T = 0.1$ K. Precession images for $\mathbf{Zn}_{0.56}$ at $T = 300$ K and $T = 100$ K are shown in Figure 1, demonstrating the absence of a low-temperature phase transition. In $\mathbf{Zn}_{0.95}$, an off-center C_{2v} interlayer site (labeled Cu2) was observed with very low occupancy. The empirical formula for this crystal ($Cu_{3.15}Zn_{0.85}(OD)_6FBr$ assuming Cu^{2+} on the off-center interlayer site) is slightly different than the formula found via ICP-AES; we believe this 50 μm crystal may not be representative of the bulk sample. It was taken from the only batch (out of approximately 60 grown for NPD) to grow crystals in addition to powder.

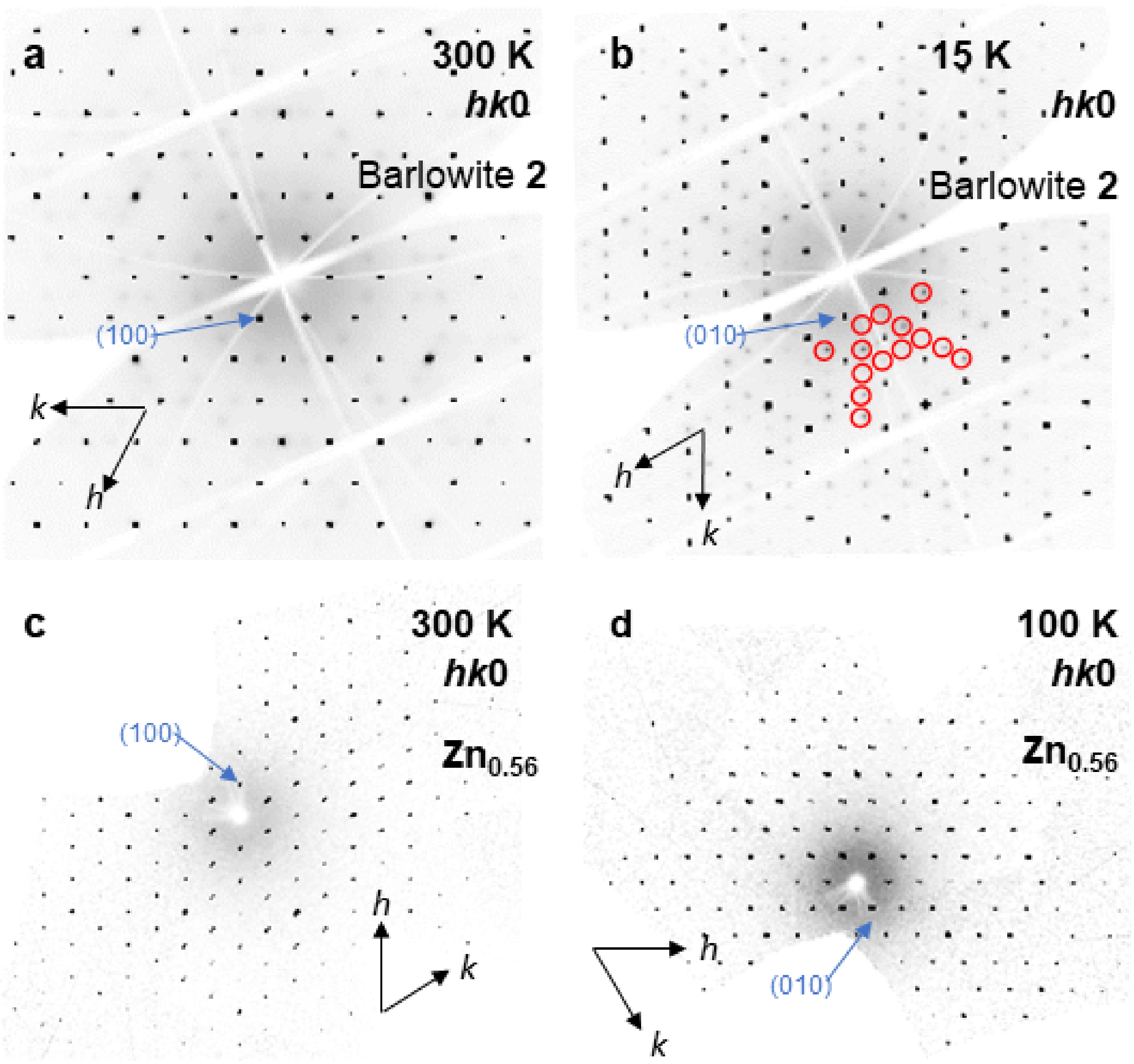

FIG. 1: SCXRD precession images of barlowite **2** in the $hk0$ zone at **a** $T = 300$ K and **b** $T = 15$ K. The superlattice peak positions expected for space group $P6_3/m$ (No. 176) are circled in red. Precession images of $\mathbf{Zn}_{0.56}$ at **c** $T = 300$ K and **d** $T = 100$ K show no superlattice peaks.

TABLE 2: Single crystal data for barlowite and Zn-substituted barlowite

**Compound**	Barlowite **1**	Barlowite **2**	$\mathbf{Zn}_{0.56}$	$\mathbf{Zn}_{0.56}$	$\mathbf{Zn}_{0.95}$
**Temperature**	17 K	15 K	300 K	100 K	100 K
**Incident Radiation** (Å)	0.41328	0.41328	0.41328	0.41328	0.7288
**Synchrotron**	APS	APS	APS	APS	ALS
Empirical Formula	$Cu_{3.99}(OD)_6FBr$	$Cu_8(OH)_{12}F_2Br_2$	$Cu_{6.8}Zn_{1.17}(OH)_{12}F_2Br_2$	$Cu_{6.97}Zn_{1.04}(OH)_{12}F_2Br_2$	$Cu_{6.3}Zn_{1.71}(OD)_{12}F_2Br_2$
Formula Weight (g·mol^{-1})	460.36	910.39	910.47	912.77	926.07
Crystal System	Orthorhombic	Hexagonal	Hexagonal	Hexagonal	Hexagonal
Space Group	*Pnma*	$P6_3/m$	$P6_3/mmc$	$P6_3/mmc$	$P6_3/mmc$
a (Å)	11.5350(3)	13.3208(3)	6.6672(9)	6.6554(2)	6.6662(2)
b (Å)	9.2601(2)	13.3208(3)	6.6672(9)	6.6554(2)	6.6662(2)
c (Å)	6.6604(2)	9.2662(3)	9.3109(12)	9.2885(2)	9.2899(4)
Volume (Å^3)	711.43(3)	1423.94(8)	358.43(11)	356.31(2)	357.52(3)
Z	4	4	1	1	1
Density (calc.) (g·cm^3)	4.298	4.247	4.218	4.254	4.301
Absorption Coeff. (mm^{-1})	3.918	3.924	3.938	3.969	18.777
F(000)	855	1712	428	429	430
Crystal Size (mm^3)	$0.03 \times 0.04 \times 0.045$	$0.035 \times 0.115 \times 0.115$	$0.01 \times 0.04 \times 0.04$	$0.01 \times 0.04 \times 0.04$	$0.05\times 0.05 \times 0.057$
θ range (°)	2.419–20.148	2.419–20.146	2.413–21.997	2.420–22.056	2.248–37.193
Index ranges	$-17 \leq h \leq 17$, $-12 \leq k \leq 13$, $-9 \leq l \leq 9$	$-21 \leq h \leq 21$, $-21 \leq k \leq 20$, $-14 \leq l \leq 14$	$-12 \leq h \leq 6$, $-9 \leq k \leq 12$, $-16 \leq l \leq 16$	$-9 \leq h \leq 12$, $-12 \leq k \leq 6$, $-16 \leq l \leq 16$	$-11 \leq h \leq 11$, $-11 \leq k \leq 10$, $-15 \leq l \leq 15$
Reflections coll./unique	25131/1276	56849/2191	14002/467	21511/464	7016/366
Completeness to θ_{max}	0.984	1.000	1.000	1.000	0.997
T_{min}, T_{max}	0.6950, 0.7445	0.661, 0.875	0.5889, 0.7447	0.6032, 0.7447	0.5705, 0.7474
R_{int}	0.0424	0.0575	0.1070	0.0861	0.0413
Goodness-of-fit on F^2	1.335	1.184	1.118	1.182	1.234
Data/parameters/restraints	1276/86/3	2191/111/4	467/26/1	464/26/0	366/26/1
Final R indices $[I>2\sigma(I)]^a$	R_1=0.0223, wR_2=0.0627	R_1=0.0281, wR_2=0.0960	R_1=0.0312, wR_2=0.0715	R_1=0.0211, wR_2=0.0560	R_1=0.0203, wR_2=0.0433
R indices (all data)^a	R_1=0.0230, wR_2=0.0628	R_1=0.0295, wR_2=0.0968	R_1=0.0336, wR_2=0.0733	R_1=0.0215, wR_2=0.0563	R_1=0.0208, wR_2=0.0438
$\Delta\rho_{max}$, $\Delta\rho_{min}$ (e·Å^{-3})	0.853, -0.676	1.664, -1.699	1.034, -0.967	0.878, -1.354	0.881, -0.658

$^a R_1 = \sum ||F_o| - |F_c|| / \sum |F_o|$, $wR_2 = [\sum w(F_o{}^2 - F_c{}^2)^2 / \sum (F_o{}^2)^2]^{1/2}$

TABLE 3: Crystallographic data for barlowite from synchrotron SCXRD. U_{eq} is defined as 1/3 of the trace of the orthogonalized U_{IJ} tensor

	**Atom**	**Wyckoff Position**	**x**	**y**	**z**	$\mathbf{U}_{eq}$ (Å^2)	**Occ.**
Barlowite	Cu1	$4a$	0.5	0.5	0.5	0.00652(13)	1
**1**	Cu2	$8d$	0.25048(3)	0.50445(4)	0.75155(5)	0.00584(11)	1
$T = 17$ K	Cu3	$4c$	0.3137(4)	0.75	0.5550(10)	0.0058(16)	0.147(5)
Pnma	Cu4	$4c$	0.3711(2)	0.75	0.4998(3)	0.0029(7)	0.327(5)
	Cu5	$4c$	0.31474(11)	0.75	0.4432(3)	0.0033(4)	0.515(5)
	Br1	$4c$	0.16663(4)	0.75	0.99838(6)	0.00522(11)	1
	F1	$4c$	-0.0001(2)	0.75	0.4974(4)	0.0077(5)	1
	O1	$8d$	0.20196(18)	0.5918(2)	0.4995(3)	0.0051(4)	1
	O2	$8d$	0.10193(18)	0.4073(2)	0.8025(3)	0.0049(4)	1
	O3	$8d$	0.40024(18)	0.5909(2)	0.6971(3)	0.0055(4)	1
	D1	$8d$	0.1400(30)	0.6430(50)	0.5020(60)	0.008	1
	D2	$8d$	0.0690(30)	0.3610(40)	0.7060(50)	0.007	1
	D3	$8d$	0.4310(40)	0.6410(40)	0.7930(50)	0.008	1
Barlowite	Cu1	$12i$	0.50001(2)	0.25046(2)	0.50000(2)	0.00590(11)	1
**2**	Cu2	$12i$	0.24982(2)	0.25019(2)	0.49711(2)	0.00562(11)	1
$T = 15$ K	Cu3	$6h$	0.62861(7)	0.31428(7)	0.75	0.00303(14)	1/3
$P6_3/m$	Cu4	$6h$	0.31422(10)	0.18552(10)	0.25	0.0058(3)	0.2396(19)
	Cu5	$6h$	0.31438(5)	0.12857(6)	0.25	0.00167(18)	0.4274(18)
	Cu6	$6h$	0.37143(8)	0.18552(7)	0.25	0.0031(2)	0.3340(18)
	Br1	$6h$	0.33332(2)	0.16618(2)	0.75	0.00386(11)	1
	Br2	$2d$	0.66667	0.33333	0.25	0.00388(11)	1
	F1	$6h$	0.50082(9)	0.50085(9)	0.25	0.0101(3)	1
	F2	$2a$	0	0	0.25	0.0100(4)	1
	O1	$12i$	0.60111(9)	0.39932(9)	0.59179(11)	0.00494(19)	1
	O2	$12i$	0.39890(9)	0.10113(9)	0.40759(10)	0.00473(18)	1
	O3	$12i$	0.39919(9)	0.29845(9)	0.40882(11)	0.00491(18)	1
	O4	$12i$	0.20187(9)	0.10024(9)	0.40823(11)	0.00471(19)	1
	H1	$12i$	0.5527(18)	0.4272(19)	0.6280(30)	0.007	1
	H2	$12i$	0.4502(18)	0.0720(20)	0.3770(30)	0.007	1
	H3	$12i$	0.4280(20)	0.3754(14)	0.3750(30)	0.007	1
	H4	$12i$	0.1243(15)	0.0516(18)	0.3700(30)	0.007	1

TABLE 4: Crystallographic data for Zn-substituted barlowite from synchrotron SCXRD. U_{eq} is defined as 1/3 of the trace of the orthogonalized U_{IJ} tensor

	**Atom**	**Wyckoff Position**	**x**	**y**	**z**	**U_{eq} (Å^2)**	**Occ.**
**Zn**$_{0.56}$	Cu1	6*g*	05	0.5	0.5	0.00996(12)	1
$T = 300$ K	Cu2	12*j*	0.3718(12)	0.7440(20)	0.25	0.0067(12)	0.127(10)
*P*6$_3$/*mmc*	Zn1	2*c*	0.33333	0.66667	0.25	0.0096(7)	0.62(3)
APS	Br1	2*d*	0.33333	0.66667	0.75	0.01500(13)	1
$Cu_{3.41}Zn_{0.59}(OH)_6FBr$	F1	2*b*	0	1	0.25	0.0187(6)	1
	O1	12*k*	0.20203(9)	0.79797(9)	0.40785(12)	0.0095(2)	1
	H1	12*k*	0.1357(16)	0.8643(16)	0.3720(20)	0.010	1
**Zn**$_{0.56}$	Cu1	6*g*	0.5	0	0.5	0.00480(9)	1
$T = 100$ K	Cu2	12*j*	0.633(10)	0.3687(10)	0.25	0.0046(8)	0.162(10)
*P*6$_3$/*mmc*	Zn1	2*c*	0.66667	0.33333	0.25	0.0023(7)	0.52(3)
APS	Br1	2*d*	0.66667	0.33333	0.75	0.005747(9)	1
$Cu_{3.50}Zn_{0.50}(OH)_6FBr$	F1	2*b*	1	0	0.25	0.0085(3)	1
	O1	12*k*	0.79823(6)	0.20177(6)	0.40761(8)	0.00441(15)	1
	H1	12*k*	0.8630(20)	0.1370(20)	0.3726(19)	0.007	1
**Zn**$_{0.95}$	Cu1	6*g*	0.5	0.5	0.5	0.00525(9)	1
$T = 100$ K	Cu2	12*j*	0.6340(60)	0.2690(120)	0.75	0.009(5)	0.049(14)
*P*6$_3$/*mmc*	Zn1	2*c*	0.66667	0.33333	0.75	0.0043(5)	0.85(4)
ALS	Br1	2*d*	0.33333	0.66667	0.75	0.00787(10)	1
$Cu_{3.15}Zn_{0.85}(OD)_6FBr$	F1	2*b*	1	1	0.75	0.0091(4)	1
	O1	12*k*	0.79792(9)	0.59584(18)	0.59261(11)	0.00555(17)	1
	D1	24*l*	0.8619(16)	0.7240(30)	0.6320(20)	0.008	1

TABLE 5: Anisotropic displacement parameters from synchrotron SCXRD. The anisotropic displacement factor exponent takes the form: $-2\pi^2[h^2a^{*2}U_{11} + 2hka^*b^*U_{12} + ...]$

	**Atom**	$\mathbf{U}_{11}$ (Å^2)	$\mathbf{U}_{22}$ (Å^2)	$\mathbf{U}_{33}$ (Å^2)	$\mathbf{U}_{23}$ (Å^2)	$\mathbf{U}_{13}$ (Å^2)	$\mathbf{U}_{12}$ (Å^2)
Barlowite	Cu1	0.0037(2)	0.012(1)	0.0037(2)	0.00040(17)	0.00012(15)	0.00252(17)
**1**	Cu2	0.00385(17)	0.01022(19)	0.00346(17)	0.00163(12)	0.00005(11)	-0.00144(11)
$T = 17$ K	Cu3	0.006(2)	0.0033(17)	0.008(3)	0	0.0002(16)	0
Pnma	Cu4	0.0042(14)	0.0012(7)	0.0034(8)	0	0.0000(7)	0
	Cu5	0.0042(7)	0.0016(4)	0.0043(9)	0	0.0009(4)	0
	Br1	0.00611(17)	0.00373(18)	0.00583(18)	0	-0.00032(11)	0
	F1	0.0070(11)	0.0095(12)	0.0066(11)	0	-0.0004(8)	0
	O1	0.0051(8)	0.0052(9)	0.0050(8)	0.0000(6)	0.0002(6)	0.0015(7)
	O2	0.0053(8)	0.0047(9)	0.0049(8)	-0.0012(7)	-0.0005(7)	-0.0005(6)
	O3	0.0050(8)	0.0061(9)	0.0054(8)	-0.0015(7)	0.0002(7)	-0.0011(7)
Barlowite	Cu1	0.00267(13)	0.00294(13)	0.01208(16)	-0.00143(6)	-0.00238(6)	0.00140(8)
**2**	Cu2	0.00289(13)	0.00297(13)	0.01116(16)	-0.00136(6)	0.00089(6)	0.00158(8)
$T = 15$ K	Cu3	0.0049(3)	0.0025(4)	0.0022(3)	0	0	0.0023(3)
*P*6$_3$/*m*	Cu4	0.0042(6)	0.0050(6)	0.0063(5)	0	0	0.0008(4)
	Cu5	0.0021(3)	0.0034(3)	0.0003(2)	0	0	0.0020(3)
	Cu6	0.0047(4)	0.0032(4)	0.0024(3)	0	0	0.0027(3)
	Br1	0.00431(13)	0.00439(13)	0.00299(14)	0	0	0.00226(7)
	Br2	0.00428(13)	0.00428(13)	0.00309(16)	0	0	0.00214(7)
	F1	0.0081(5)	0.0092(5)	0.0130(8)	0	0	0.0044(4)
	F2	0.0082(6)	0.0082(6)	0.0135(11)	0	0	0.0041(3)
	O1	0.0036(4)	0.0042(4)	0.0067(4)	-0.0013(3)	0.0001(3)	0.0017(3)
	O2	0.0034(4)	0.0043(4)	0.0065(4)	-0.0010(3)	0.0001(3)	0.0019(3)
	O3	0.0043(4)	0.0043(4)	0.0067(4)	0.0014(3)	0.0011(3)	0.0026(3)
	O4	0.0039(4)	0.0033(4)	0.0065(4)	-0.0002(3)	-0.0014(3)	0.0015(3)
**Zn**$_{0.56}$	Cu1	0.00789(16)	0.00827(14)	0.01360(19)	0.00173(4)	0.00347(7)	0.00395(8)
$T = 300$ K	Cu2	0.0060(14)	0.008(3)	0.0072(9)	0	0	0.0038(15)
*P*6$_3$/*mmc*	Zn1	0.0116(11)	0.0116(11)	0.0055(4)	0	0	0.0058(5)
	Br1	0.01678(15)	0.01678(15)	0.01144(19)	0	0	0.00839(8)
	F1	0.0139(7)	0.0139(7)	0.0283(18)	0	0	0.0070(4)
	O1	0.0084(3)	0.0084(5)	0.0116(4)	0.00059(16)	-0.00059(16)	0.0042(3)
**Zn**$_{0.56}$	Cu1	0.00340(10)	0.00329(11)	0.00769(14)	0.00183(4)	0.00091(2)	0.00164(6)
$T = 100$ K	Cu2	0.0045(8)	0.0045(8)	0.0030(5)	0	0	0.0010(6)
*P*6$_3$/*mmc*	Zn1	0.0022(10)	0.0022(10)	0.0025(3)	0	0	0.0011(5)
	Br1	0.00642(10)	0.00642(10)	0.00440(13)	0	0	0.00321(5)
	F1	0.0072(5)	0.0072(5)	0.001120(9)	0	0	0.0036(2)
	O1	0.0045(2)	0.0045(2)	0.0060(3)	-0.00042(10)	0.00042(10)	0.0025(2)
**Zn**$_{0.95}$	Cu1	0.00426(11)	0.00426(11)	0.00698(14)	0.00105(3)	-0.00105(3)	0.00195(9)
$T = 100$ K	Cu2	0.007(4)	0.021(14)	0.004(3)	0	0	0.011(7)
*P*6$_3$/*mmc*	Zn1	0.0047(6)	0.0047(6)	0.0036(4)	0	0	0.0023(3)
	Br1	0.00830(12)	0.00830(12)	0.00702(16)	0	0	0.00415(6)
	F1	0.0069(6)	0.0069(6)	0.0136(12)	0	0	0.0034(3)
	O1	0.0051(3)	0.0049(4)	0.0065(3)	-0.0005(3)	-0.00023(16)	0.0025(2)

TABLE 6: Selected bond angles relating to the kagome Cu's extracted from single crystal refinements of barlowite and Zn-substituted barlowite

	Barlowite **1**		Barlowite **2**
**Temperature**	17 K		15 K
**Space Group**	*Pnma*		$P6_3/m$
Cu1–O2–Cu2	115.97(10)°	Cu1–O1–Cu1	117.20(5)°
Cu1–O3–Cu2	118.45(11)°	Cu1–O2–Cu2	116.44(5)°
Cu2–O1–Cu2	117.30(11)°	Cu1–O3–Cu2	118.04(5)°
Cu1–O2–Cu5	107.18(11)°	Cu2–O4–Cu2	117.24(5)°
Cu1–O2–Cu3	92.19(15)°	Cu1–O1–Cu3 (1)	107.27(5)°
Cu1–O2–Cu4	88.35(10)°	Cu1–O1–Cu3 (2)	91.83(6)°
Cu2–O1–Cu3	108.1(2)°	Cu1–O1–Cu3 (3)	88.43(5)°
Cu2–O1–Cu4	92.87(10)°	Cu2–O2–Cu6	108.04(5)°
Cu2–O1–Cu5	89.60(10)°	Cu2–O2–Cu4	92.49(5)°
		Cu2–O2–Cu5	89.17(4)°

	$\mathbf{Zn}_{0.56}$	$\mathbf{Zn}_{0.56}$	$\mathbf{Zn}_{0.95}$
**Temperature**	300 K	100 K	100 K
**Space Group**	$P6_3/mmc$	$P6_3/mmc$	$P6_3/mmc$
Cu1–O1–Cu1	117.01(5)°	116.85(4)°	116.92(5)°
Cu1–O1–Zn1	95.85(4)°	95.76(2)°	95.85(3)°
Cu1–O1–Cu2 (1)	107.7(4)°	106.6(3)°	105.7(19)°
Cu1–O1–Cu2 (2)	92.1(2)°	92.27(9)°	92.6(5)°
Cu1–O1–Cu2 (3)	88.6(2)°	89.1(2)°	89.8(12)°

TABLE 7: Selected bond distances in Å extracted from single crystal refinements of barlowite and Zn-substituted barlowite

**Temperature**<br>**Space Group**	Barlowite **1**<br>17 K<br>*Pnma*		Barlowite **2**<br>15 K<br>$P6_3/m$		$\mathbf{Zn}_{0.56}$<br>300 K<br>$P6_3/mmc$	$\mathbf{Zn}_{0.56}$<br>100 K<br>$P6_3/mmc$	$\mathbf{Zn}_{0.95}$<br>100 K<br>$P6_3/mmc$
Cu1–Cu2 (1)	3.3306(4)	Cu1–Cu1	3.3300(5)	Cu1–Cu1	3.3336(5)	3.32770(11)	3.33310(11)
Cu1–Cu2 (2)	3.3299(4)	Cu1–Cu2 (1)	3.3297(4)	Cu1–O1	1.9548(6)	1.9529(4)	1.9555(6)
Cu2–Cu2	3.33124(11)	Cu1–Cu2 (2)	3.3310(5)	Cu1–Br1	3.0204(3)	3.01388(6)	3.01614(9)
Cu1–Br1	3.0090(3)	Cu2–Cu2	3.3307(3)	Zn1–O1	2.1117(11)	2.1079(8)	2.1058(11)
Cu2–Br1	2.9679(5)	Cu1–Br1	3.0106(3)	Cu2–O1 (1)	1.996(3)	1.998(2)	2.002(13)
Cu1–O2	1.962(2)	Cu1–Br2	3.0104(3)	Cu2–O1 (2)	1.996(2)	1.998(2)	2.002(13)
Cu1–O3	1.938(2)	Cu2–Br1	2.9836(3)	Cu2–O1 (3)	2.452(10)	2.418(10)	2.39(5)
Cu2–O1	1.946(2)	Cu1–O1	1.9522(10)	Cu2–Cu2	0.77(2)	0.71(2)	0.65(12)
Cu2–O2	1.965(2)	Cu1–O2	1.9557(10)	Cu1–Cu2 (1)	2.759(8)	2.772(7)	2.79(4)
Cu2–O3	1.938(2)	Cu1–O3	1.9455(15)	Cu1–Cu2 (2)	3.192(10)	3.167(5)	3.15(6)
Cu3–O1	1.986(4)	Cu2–O2	1.9614(10)	Cu1–Cu2 (3)	3.192(10)	3.167(5)	3.15(6)
Cu3–O2	2.429(6)	Cu2–O3	1.9397(10)	Cu1–Zn1	3.0204(3)	3.01388(6)	3.01614(8)
Cu3–O3	2.016(4)	Cu2–O4	1.9494(10)				
Cu4–O1	2.440(3)	Cu3–O1 (1)	1.9954(12)				
Cu4–O2	1.986(3)	Cu3–O1 (2)	1.9938(12)				
Cu4–O3	2.003(3)	Cu3–O1 (3)	2.4448(15)				
Cu5–O1	1.995(2)	Cu4–O2	2.4365(15)				
Cu5–O2	1.981(2)	Cu4–O3	2.0018(13)				
Cu5–O3	2.450(3)	Cu4–O4	1.9949(13)				
Cu3–Cu4	0.757(6)	Cu4–Cu5	0.7598(16)				
Cu3–Cu5	0.745(7)	Cu4–Cu6	0.7621(17)				
Cu4–Cu5	0.752(3)	Cu5–Cu6	0.7593(12)				
Cu1–Cu3	3.180(4)	Cu1–Cu3 (1)	2.7509(5)				
Cu1–Cu4	2.7514(13)	Cu1–Cu3 (2)	3.1771(9)				
Cu1–Cu5	3.1732(9)	Cu1–Cu3 (3)	3.1789(6)				
Cu2–Cu3	3.191(5)	Cu1–Cu4	3.1779(10)				
Cu2–Cu4	3.2020(15)	Cu1–Cu5	3.1784(5)				
Cu2–Cu5	2.7834(11)	Cu1–Cu6	2.7508(6)				
		Cu2–Cu4	2.7313(10)				
		Cu2–Cu5	3.1624(7)				
		Cu2–Cu6	3.1630(9)				

III. POWDER X-RAY AND NEUTRON DIFFRACTION OF BARLOWITE

Synchrotron powder X-ray diffraction (PXRD) was measured on barlowite **1** and **2**, and neutron powder diffraction (NPD) was measured on barlowite **1** at a variety of temperatures. Rietveld co-refinements of the PXRD and NPD data were performed on barlowite **1** (Figures 2–4; Table 8). For barlowite **1** below the phase transition, a mixture of two phases was found in the synchrotron PXRD data—both the low-temperature orthorhombic phase (*Pnma*) and the high-temperature hexagonal phase ($P6_3/mmc$). The hexagonal phase fraction refined to approximately 30%. This was not observed in the NPD data, so only the orthorhombic phase was refined.

Rietveld refinements of the PXRD data of barlowite **2** were performed (Figures 5–6; Table 9). Superlattice peaks due to space group $P6_3/m$ are not visible (Figure 1c in the main text and Figures 5–6), suggesting that they are too weak to detect when crystals are ground into a powder.[68] This supports the claim that barlowite **2** is not orthorhombic. In addition, an analysis of orthorhombic distortion was performed on the PXRD data of barlowite **1** and **2** (protonated and deuterated of each) at the lowest temperature measured, $T = 13$ K. The orthorhombic splitting $(b - a)/(b + a)$ was calculated for three in-plane peaks ([1 1 0], [2 1 0], and [2 2 0], using the notation of the high-temperature space group), where a and b are the positions in q (Å^{-1}) of the left- and right-hand peaks, respectively. For barlowite **2**, these positions were picked within the peak (which does not split and exhibits very little to no broadening in FWHM from $T = 300$ K to $T = 13$ K) and represent an upper limit of possible splitting. The average distortion for barlowite **1** is 0.00137, while the upper limit for the average distortion in barlowite **2** is 20 times smaller (0.000072), demonstrating that barlowite **2** remains hexagonal.

Selected bond distances and angles for both are tabulated in Tables 10–11.

TABLE 8: Crystallographic data for barlowite **1** elucidated through Rietveld co-refinements of synchrotron PXRD (λ = 0.412728 Å) and NPD (λ = 2.0775 Å) data

	Atom	Wyckoff Position	x	y	z	U_{iso}	Occ.
Barlowite **1**[a]	Cu1	4*a*	0.5	0.5	0.5	0.00376(8)	1
Pnma	Cu2	8*d*	0.25116(7)	0.50928(6)	0.75278(10)	0.00376(8)	1
PXRD: $T = 13$ K	Cu3	4*c*	0.31562(97)	0.75	0.56826(167)	0.00376(8)	0.112(2)
NPD: $T = 2$ K	Cu4	4*c*	0.37373(184)	0.75	0.48554(332)	0.00376(8)	0.054(2)
a = 11.525898(17) Å	Cu5	4*c*	0.31614(14)	0.75	0.44473(22)	0.00376(8)	0.833(3)
b = 9.269405(14) Å	Br1	4*c*	0.16841(6)	0.75	0.99687(11)	0.00387(11)	1
c = 6.678477(10) Å	F1	4*c*	0.00142(31)	0.75	0.49593(55)	0.00534(38)	1
V = 713.517(3) Å^3	O1	8*d*	0.20281(21)	0.59230(30)	0.49829(43)	0.00466(21)	1
	O2	8*d*	0.10324(26)	0.40642(29)	0.80231(40)	0.00466(21)	1
	O3	8*d*	0.40043(26)	0.58930(28)	0.69559(39)	0.00466(21)	1
	D1	8*d*	0.12245(30)	0.63656(47)	0.49703(80)	0.01826(23)	1
	D2	8*d*	0.06269(44)	0.36961(42)	0.68540(65)	0.01826(23)	1
	D3	8*d*	0.43942(43)	0.63465(44)	0.80613(60)	0.01826(23)	1
Barlowite **1**[b]	Cu1	4*a*	0.5	0.5	0.5	0.00308(10)	1
Pmna	Cu2	8*d*	0.25131(7)	0.50952(6)	0.75283(10)	0.00308(10)	1
$T = 100$ K	Cu3	4*c*	0.31331(100)	0.75	0.56832(188)	0.00308(10)	0.108(2)
a = 11.530342(17) Å	Cu4	4*c*	0.38856(988)	0.75	0.48223(1744)	0.00308(10)	0.009(2)
b = 9.279475(14) Å	Cu5	4*c*	0.31635(13)	0.75	0.44392(58)	0.00308(10)	0.883(3)
c = 6.684333(10) Å	Br1	4*c*	0.16879(7)	0.75	0.99687(11)	0.00465(15)	1
V = 715.194(2) Å^3	F1	4*c*	0.00365(34)	0.75	0.49435(58)	0.00582(51)	1
	O1	8*d*	0.20247(23)	0.59238(33)	0.49839(45)	0.00371(28)	1
	O2	8*d*	0.10317(28)	0.40659(31)	0.80227(42)	0.00371(28)	1
	O3	8*d*	0.40069(28)	0.58856(30)	0.69433(42)	0.00371(28)	1
	D1	8*d*	0.12289(45)	0.63665(69)	0.49411(106)	0.01790(34)	1
	D2	8*d*	0.06416(60)	0.36949(60)	0.68422(90)	0.01790(34)	1
	D3	8*d*	0.43887(60)	0.63494(62)	0.80491(81)	0.01790(34)	1
Barlowite **1**[c]	Cu1	6*g*	0.5	0	0.5	0.00938(8)	1
$P6_3/mmc$	Cu2	6*h*	0.62880(24)	0.25760(48)	0.25	0.00938(8)	1/3
$T = 300$ K	Br1	2*c*	0.66667	0.33333	0.75	0.01369(12)	1
a = 6.679488(26) Å	F1	2*b*	1	0	0.75	0.01578(42)	1
c = 9.295559(11) Å	O1	12*k*	0.79733(6)	0.20267(6)	0.40831(8)	0.00875(21)	1
V = 359.164(2) Å^3	D1	12*k*	0.87465(9)	0.12535(9)	0.36658(10)	0.02434(26)	1

[a]Overall GOF = 2.14, Overall w*R* = 8.55%.
PXRD: w*R* = 11.28%; $P6_3/mmc$ phase 0.26366(167) wt.%. NPD: w*R* = 3.35%.
[b]Overall GOF = 2.48, Overall w*R* = 9.73%.
PXRD: w*R* = 11.21%; $P6_3/mmc$ phase 0.31738(140) wt.%. NPD: w*R* = 4.68%.
[c]Overall GOF = 2.09, Overall w*R* = 9.29%.
PXRD: w*R* = 11.39%. NPD: w*R* = 4.41%.

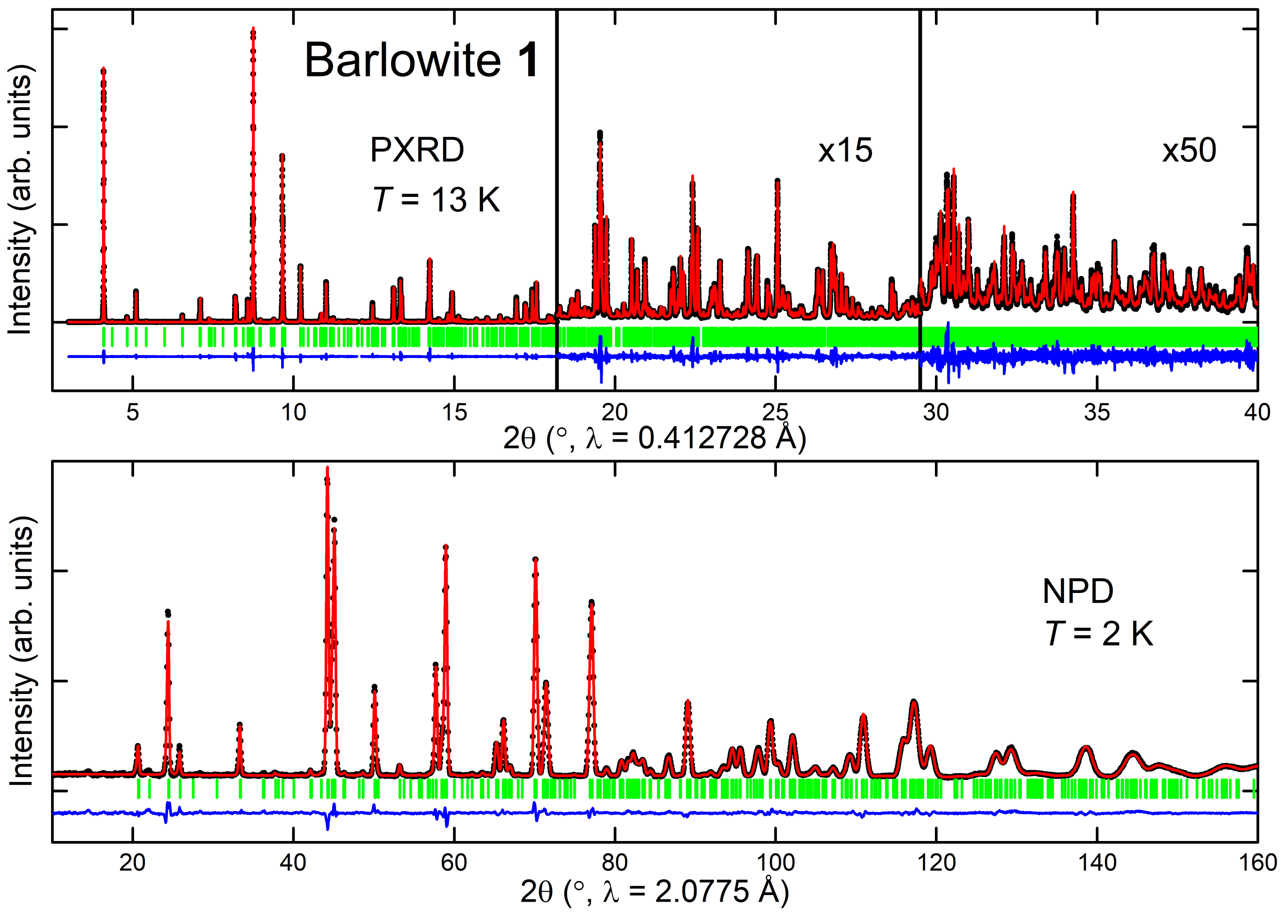

FIG. 2: Rietveld co-refinement of synchrotron PXRD (top) and NPD (bottom) data of barlowite **1** at $T = 13$ K and 2 K, respectively. Observed (black), calculated (red), and difference (blue) plots are shown, and Bragg reflections are indicated by green tick marks.

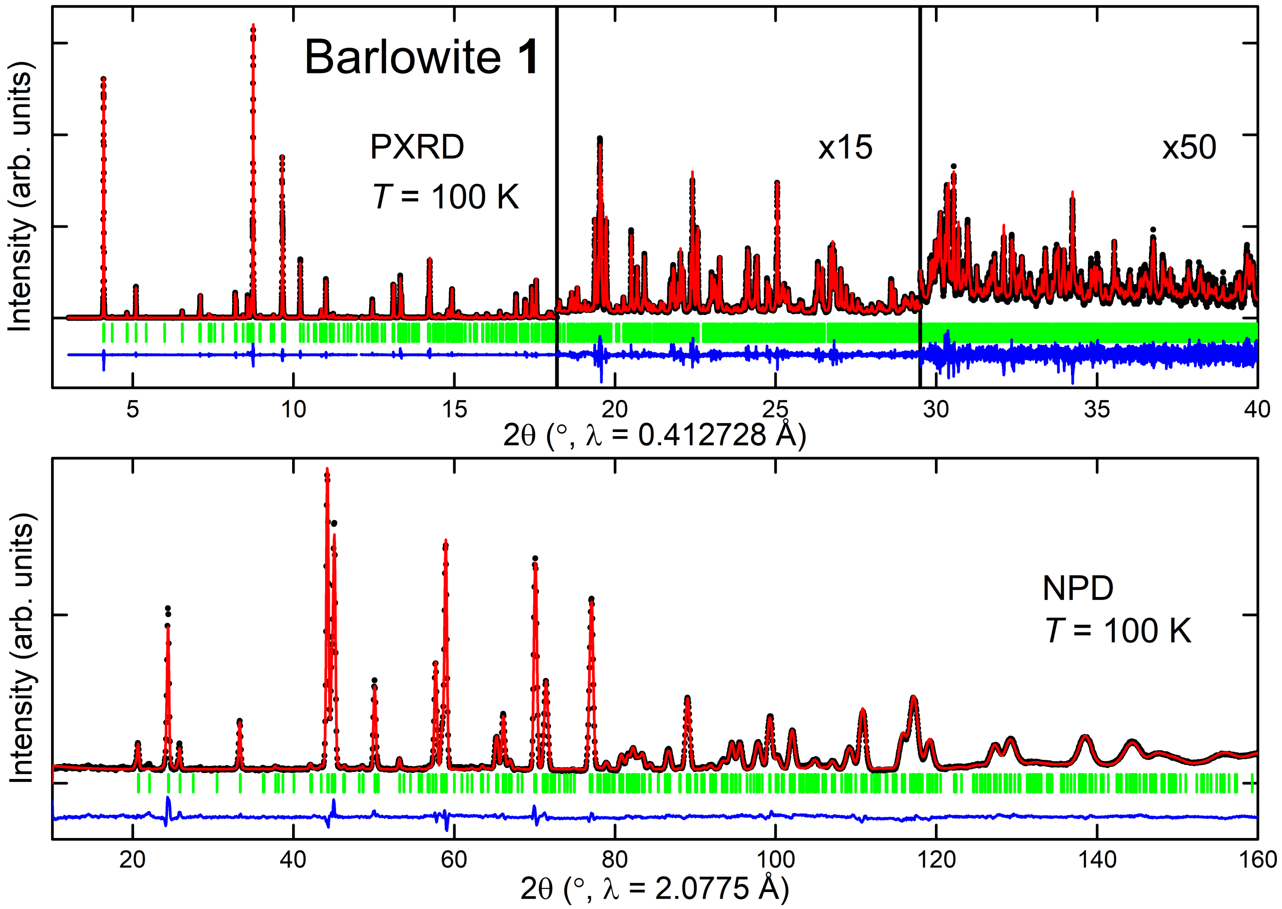

FIG. 3: Rietveld co-refinement of synchrotron PXRD (top) and NPD (bottom) data of barlowite **1** at $T = 100$ K. Observed (black), calculated (red), and difference (blue) plots are shown, and Bragg reflections are indicated by green tick marks.

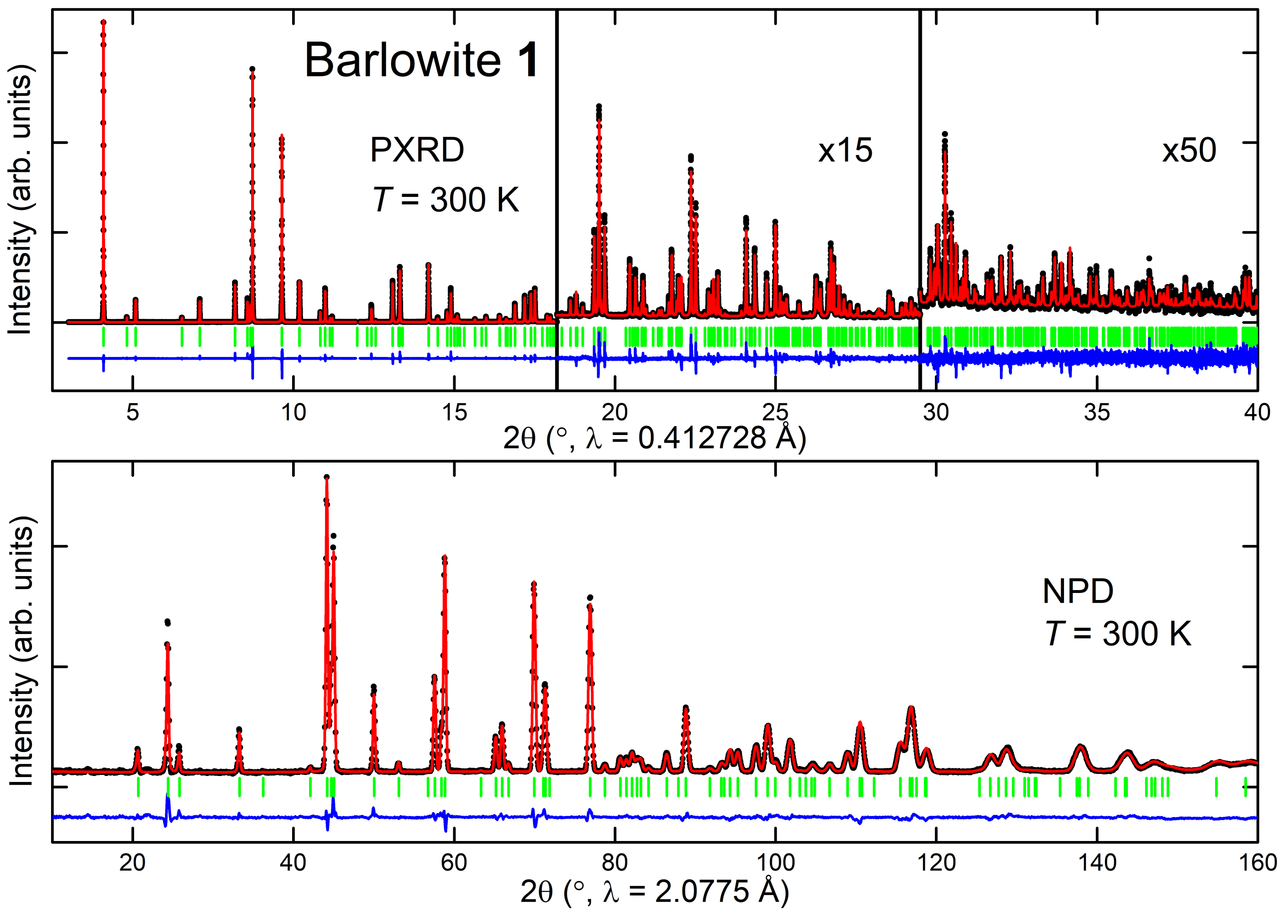

FIG. 4: Rietveld co-refinement of synchrotron PXRD (top) and NPD (bottom) data of barlowite **1** at $T = 300$ K. Observed (black), calculated (red), and difference (blue) plots are shown, and Bragg reflections are indicated by green tick marks.

TABLE 9: Crystallographic data for barlowite **2** elucidated through Rietveld refinements of synchrotron PXRD data (λ = 0.412728 Å) in $P6_3/m$

	Atom	Wyckoff Position	x	y	z	U_{iso}	Occ.
Barlowite **2**[a]	Cu1	12*i*	0.49935(40)	0.24984(31)	0.50333(13)	0.00777(7)	1
$T = 14$ K	Cu2	12*i*	0.24957(37)	0.25014(42)	0.49603(11)	0.00777(7)	1
a = 13.332954(89) Å	Cu3	6*h*	0.62989(96)	0.31106(177)	0.75	0.00777(7)	1/3
c = 9.270885(9) Å	Cu4	6*h*	0.31220(249)	0.18711(262)	0.25	0.00777(7)	0.267(14)
V = 1427.265(11) Å^3	Cu5	6*h*	0.31576(192)	0.13144(81)	0.25	0.00777(7)	0.419(11)
	Cu6	6*h*	0.37486(186)	0.18446(231)	0.25	0.00777(7)	0.314(17)
	Br1	6*h*	0.33259(43)	0.16575(24)	0.75	0.00592(9)	1
	Br2	2*d*	0.66667	0.33333	0.25	0.00592(9)	1
	F1	6*h*	0.50038(118)	0.50112(121)	0.25	0.01015(51)	1
	F2	2*a*	0	0	0.25	0.01015(51)	1
	O1	12*i*	0.59890(108)	0.39839(99)	0.59287(75)	0.00354(32)	1
	O2	12*i*	0.40124(107)	0.10421(102)	0.40377(80)	0.00354(32)	1
	O3	12*i*	0.39914(117)	0.29885(92)	0.40558(80)	0.00354(32)	1
	O4	12*i*	0.19650(102)	0.09408(95)	0.40491(115)	0.00354(32)	1
Barlowite **2**[b]	Cu1	12*i*	0.49977(35)	0.25030(32)	0.50191(22)	0.00750(8)	1
$T = 99$ K	Cu2	12*i*	0.24966(30)	0.24971(33)	0.49685(14)	0.00750(8)	1
a = 13.333720(78) Å	Cu3	6*h*	0.63068(105)	0.30975(92)	0.75	0.00750(8)	1/3
c = 9.275651(8) Å	Cu4	6*h*	0.31108(190)	0.18579(176)	0.25	0.00750(8)	0.266(12)
V = 1428.163(10) Å^3	Cu5	6*h*	0.31742(124)	0.13032(88)	0.25	0.00750(8)	0.437(11)
	Cu6	6*h*	0.37487(175)	0.18709(172)	0.25	0.00750(8)	0.297(15)
	Br1	6*h*	0.33195(34)	0.16547(21)	0.75	0.00710(10)	1
	Br2	2*d*	0.66667	0.33333	0.25	0.00710(10)	1
	F1	6*h*	0.50082(127)	0.50249(131)	0.25	0.01180(57)	1
	F2	2*a*	0	0	0.25	0.01180(57)	1
	O1	12*i*	0.60065(204)	0.40163(201)	0.59064(108)	0.00343(32)	1
	O2	12*i*	0.39759(202)	0.10126(200)	0.40647(92)	0.00343(32)	1
	O3	12*i*	0.39976(200)	0.29853(93)	0.40726(89)	0.00343(32)	1
	O4	12*i*	0.19328(82)	0.09480(192)	0.40430(114)	0.00343(32)	1

[a]GOF = 2.64, w*R* = 12.36%. CuOHF impurity: 0.01908(24) wt.%.
[b]GOF = 1.68, w*R* = 11.41%. CuOHF impurity: 0.01620(17) wt.%.

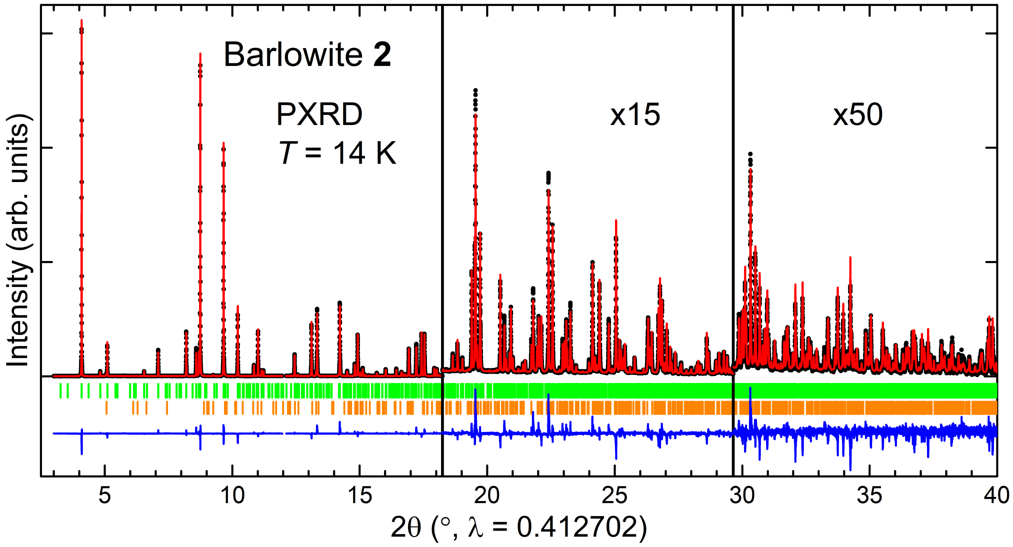

FIG. 5: Rietveld refinement of synchrotron PXRD data of barlowite **2** at $T = 14$ K. Observed (black), calculated (red), and difference (blue) plots are shown, and Bragg reflections are indicated by green tick marks. The Bragg reflections of a CuOHF impurity phase are marked with orange tick marks.

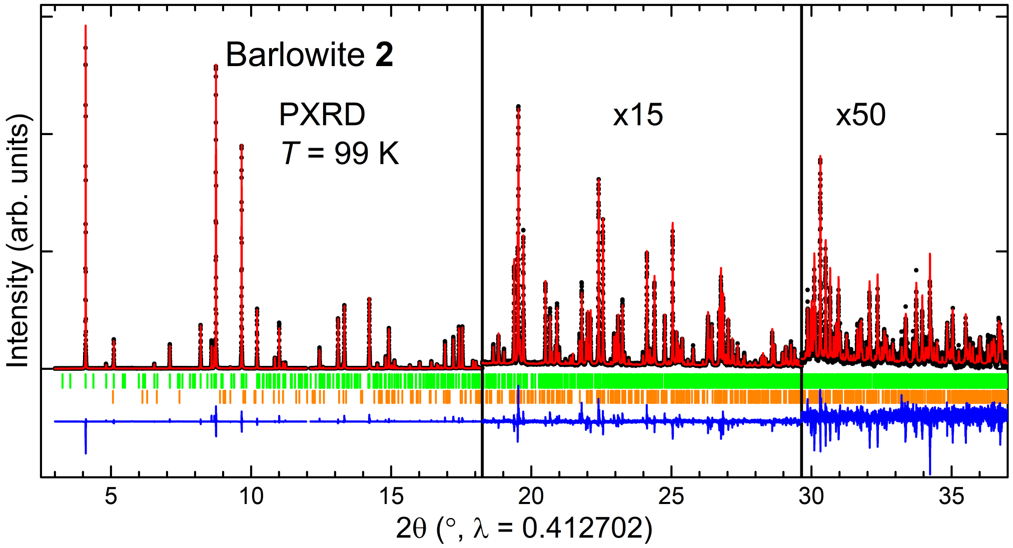

FIG. 6: Rietveld refinement of synchrotron PXRD data of barlowite **2** at $T = 99$ K. Observed (black), calculated (red), and difference (blue) plots are shown, and Bragg reflections are indicated by green tick marks. The Bragg reflections of a CuOHF impurity phase are marked with orange tick marks.

TABLE 10: Selected bond angles relating to the kagome Cu's extracted from Rietveld refinements of barlowite **1** and **2**

	Barlowite **1**	Barlowite **1**		Barlowite **1**
**Radiation**	PXRD / NPD	PXRD / NPD		PXRD / NPD
**Temperature**	13 / 2 K	100 K		300 K
**Space Group**	*Pnma*	*Pnma*		$P6_3/mmc$
Cu1–O2–Cu2	114.72(14)°	114.71(15)°	Cu1–O1–Cu1	117.51(4)°
Cu1–O3–Cu2	120.33(14)°	120.81(15)°	Cu1–O1–Cu2 (1)	107.63(3)°
Cu2–O1–Cu2	117.51(14)°	117.37(14)°	Cu1–O1–Cu2 (2)	92.24(4)°
Cu1–O2–Cu5	106.54(14)°	106.61(14)°	Cu1–O1–Cu2 (3)	88.72(4)°
Cu1–O2–Cu3	90.3(3)°	90.6(3)°		
Cu1–O2–Cu4	89.3(6)°	87(4)°		
Cu2–O1–Cu3	111.6(4)°	111.9(4)°		
Cu2–O1–Cu4	92.3(5)°	91(3)°		
Cu2–O1–Cu5	91.42(12)°	91.25(14)°		

	Barlowite **2**	Barlowite **2**
**Radiation**	PXRD	PXRD
**Temperature**	14 K	99 K
**Space Group**	$P6_3/m$	$P6_3/m$
Cu1–O1–Cu1	118.0(6)°	117.6(11)°
Cu1–O2–Cu2	114.1(6)°	116.0(10)°
Cu1–O3–Cu2	116.1(5)°	117.0(5)°
Cu2–O4–Cu2	114.2(6)°	113.0(5)°
Cu1–O1–Cu3 (1)	105.5(8)°	105.3(14)°
Cu1–O1–Cu3 (2)	89.7(8)°	89.4(11)°
Cu1–O1–Cu3 (3)	88.1(9)°	89.3(12)°
Cu2–O2–Cu6	109.1(10)°	110.1(15)°
Cu2–O2–Cu4	92.0(9)°	92.6(12)°
Cu2–O2–Cu5	89.3(8)°	90.8(12)°

TABLE 11: Selected bond distances in Å extracted from Rietveld refinements of barlowite

**Radiation** **Temperature** **Space Group**	Barlowite **1** PXRD / NPD 13 / 2 K *Pnma*	Barlowite **1** PXRD / NPD 100 K *Pnma*		Barlowite **1** PXRD / NPD 300 K $P6_3/mmc$		Barlowite **2** PXRD 14 K $P6_3/m$	Barlowite **2** PXRD 99 K $P6_3/m$
Cu1–Cu2 (1)	3.3337(8)	3.3368(8)	Cu1–Cu1	3.33974(0)	Cu1–Cu1	3.347(9)	3.337(9)
Cu1–Cu2 (2)	3.3292(8)	3.3296(8)	Cu1–O1	1.9531(5)	Cu1–Cu2 (1)	3.324(7)	3.332(8)
Cu2–Cu2	3.34377(6)	3.34697(7)	Cu1–Br1	3.01967(0)	Cu1–Cu2 (2)	3.332(9)	3.336(6)
Cu1–Br1	3.0230(5)	3.0282(6)	Cu2–O1 (1)	1.9951(13)	Cu2–Cu2	3.332(4)	3.330(3)
Cu2–Br1	2.9233(8)	3.0944(8)	Cu2–O1 (2)	1.9951(13)	Cu1–Br1	2.989(5)	3.008(5)
Cu1–O2	1.978(3)	1.978(3)	Cu2–O1 (3)	2.4428(6)	Cu1–Br2	3.041(4)	3.029(4)
Cu1–O3	1.926(3)	1.917(3)	Cu2–Cu2	0.75879(0)	Cu2–Br1	2.974(5)	3.035(5)
Cu2–O1	1.947(3)	1.950(4)	Cu1–Cu2 (1)	2.76060(0)	Cu1–O1	1.935(11)	1.96(2)
Cu2–O2	1.981(4)	1.985(4)	Cu1–Cu2 (2)	3.1867(9)	Cu1–O2	1.948(11)	1.970(19)
Cu2–O3	1.912(4)	1.913(4)	Cu1–Cu2 (3)	3.1867(9)	Cu1–O3	1.976(17)	1.95(3)
Cu3–O1	2.011(9)	1.998(9)			Cu2–O2	2.012(12)	1.96(2)
Cu3–O2	2.477(10)	2.490(11)			Cu2–O3	1.950(15)	1.95(3)
Cu3–O3	1.974(8)	1.992(9)			Cu2–O4	1.950(15)	1.99(3)
Cu4–O1	2.454(17)	2.60(10)			Cu3–O1 (1)	2.03(3)	2.08(3)
Cu4–O2	1.918(14)	1.89(8)			Cu3–O1 (2)	1.97(3)	1.98(3)
Cu4–O3	2.067(15)	2.07(8)			Cu3–O1 (3)	2.442(17)	2.46(3)
Cu5–O1	1.993(3)	1.999(3)			Cu4–O2	2.44(4)	2.45(4)
Cu5–O2	1.967(3)	1.967(4)			Cu4–O3	1.98(2)	2.002(16)
Cu5–O3	2.443(3)	2.448(4)			Cu4–O4	2.02(2)	2.020(17)
Cu3–Cu4	0.87(3)	1.05(12)			Cu4–Cu5	0.77(5)	0.79(4)
Cu3–Cu5	0.825(12)	0.832(13)			Cu4–Cu6	0.85(5)	0.84(4)
Cu4–Cu5	0.72(3)	0.88(12)			Cu5–Cu6	0.75(3)	0.76(2)
Cu1–Cu3	3.177(8)	3.198(8)			Cu1–Cu3 (1)	2.740(8)	2.754(8)
Cu1–Cu4	2.738(12)	2.65(6)			Cu1–Cu3 (2)	3.185(14)	3.200(8)
Cu1–Cu5	3.1618(11)	3.1633(11)			Cu1–Cu3 (3)	3.130(16)	3.119(12)
Cu2–Cu3	2.655(7)	2.648(7)			Cu1–Cu4	3.22(3)	3.220(18)
Cu2–Cu4	3.186(15)	3.28(9)			Cu1–Cu5	3.183(14)	3.169(9)
Cu2–Cu5	3.1261(13)	3.1314(12)			Cu1–Cu6	2.753(12)	2.746(12)
					Cu2–Cu4	3.22(3)	3.208(17)
					Cu2–Cu5	2.798(7)	2.789(7)
					Cu2–Cu6	3.201(17)	3.218(13)

IV. POWDER X-RAY AND NEUTRON DIFFRACTION OF ZN-SUBSTITUTED BARLOWITE

Rietveld co-refinements of PXRD and NPD data collected at low temperature ($T_{\mathrm{NPD}} = 3$ K and $T_{\mathrm{PXRD}} = 12$ K) and $T = 300$ K were performed on **Zn**$_{0.95}$ (Figures 7–8; Table 12). Rietveld refinements of PXRD data collected at $T = 90$ K and $T = 295$ K were performed on **Zn**$_{0.56}$ (Figures 9–10; Table 12). Selected bond angles and distances are in Tables 13–14.

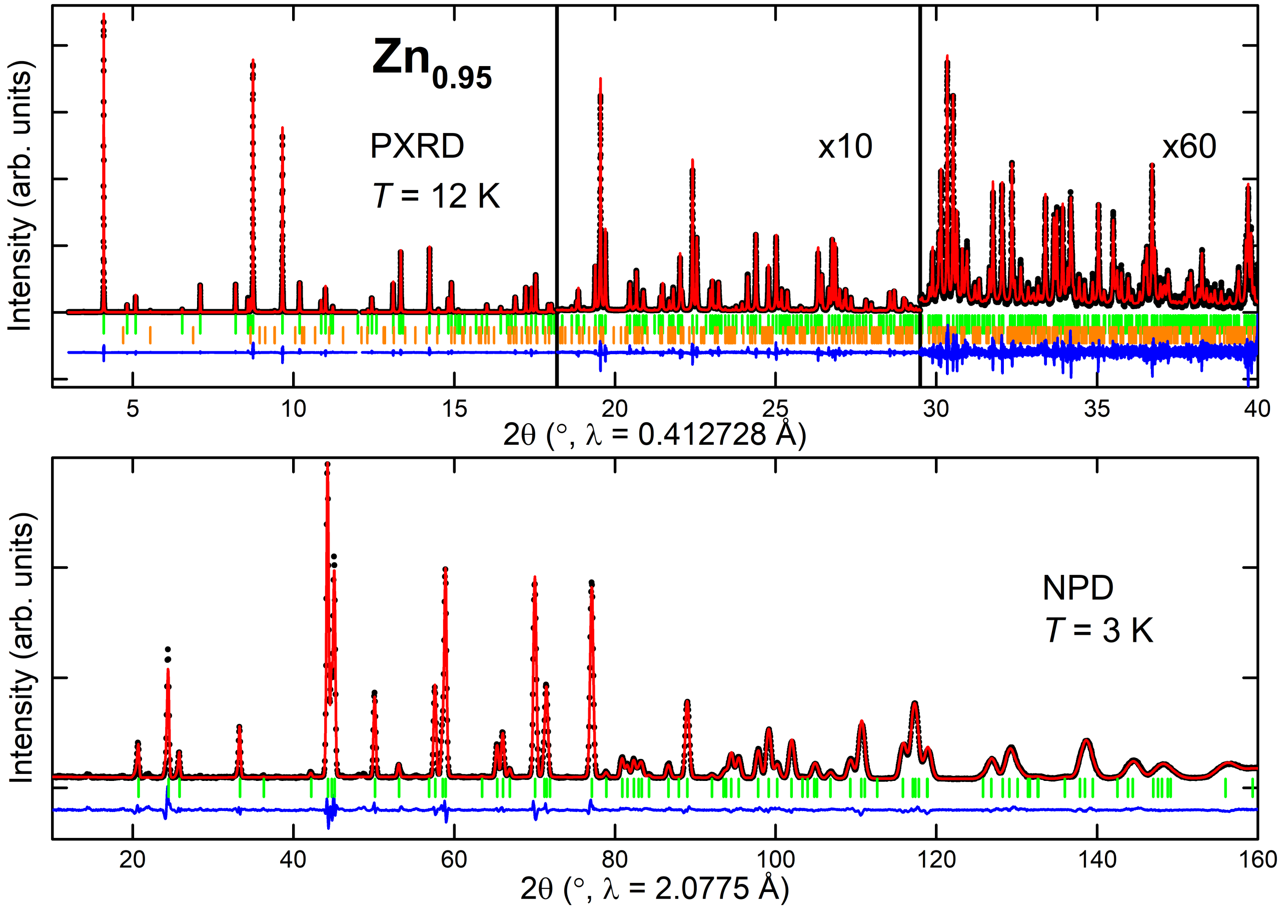

FIG. 7: Rietveld co-refinement of synchrotron PXRD (top) and NPD (bottom) data of deuterated **Zn**$_{0.95}$ at $T = 12$ K and 2 K, respectively. Observed (black), calculated (red), and difference (blue) plots are shown, and Bragg reflections are indicated by green tick marks. The Bragg reflections of a ZnOHF impurity phase are marked with orange tick marks.

TABLE 12: Crystallographic data for Zn-substituted barlowite. $\mathbf{Zn}_{0.95}$ is elucidated through Rietveld co-refinements of synchrotron PXRD (λ = 0.412728 Å) and NPD (λ = 2.0775 Å) data. $\mathbf{Zn}_{0.56}$ is elucidated through Rietveld refinements of synchrotron PXRD data (λ = 0.412702 Å), and the refined formula, not including H, is shown. Both are refined in $P6_3/mmc$

	Atom	Wyckoff Position	x	y	z	U_{iso}	Occ.
$\mathbf{Zn}_{0.95}{}^{a}$	Cu1	6*g*	0.5	0	0.5	0.00209(7)	1
PXRD: $T = 13$ K	Zn1	2d	0.66667	0.33333	0.25	0.00869(19)	1
NPD: $T = 3$ K	Br1	2*c*	0.66667	0.33333	0.75	0.00268(13)	1
a = 6.665674(33) Å	F1	2*b*	1	0	0.75	0.00414(37)	1
c = 9.295618(14) Å	O1	12*k*	0.79790(7)	0.20210(7)	0.40747(7)	0.00233(22)	1
V = 357.682(2) Å^3	D1	12*k*	0.87553(9)	0.12447(9)	0.36587(10)	0.01381(26)	1
$\mathbf{Zn}_{0.95}{}^{b}$	Cu1	6*g*	0.5	0	0.5	0.00853(7)	1
$T = 300$ K	Zn1	2*d*	0.66667	0.33333	0.25	0.01360(23)	1
a = 6.678203(31) Å	Br1	2*c*	0.66667	0.33333	0.75	0.01621(19)	1
c = 9.317876(14) Å	F1	2*b*	1	0	0.75	0.01589(39)	1
V = 359.887(2) Å^3	O1	12*k*	0.79761(6)	0.20239(6)	0.40778(7)	0.00899(21)	1
	D1	12*k*	0.87521(8)	0.12479(8)	0.36664(9)	0.02108(24)	1
$\mathbf{Zn}_{0.56}{}^{c}$	Cu1	6*g*	0.5	0	0.5	0.00162(4)	1
$T = 90$ K	Zn1	2*d*	0.66667	0.33333	0.25	0.00575(28)	0.615(6)
a = 6.660641(9) Å	Cu2	6*h*	0.63010(119)	0.26014(237)	0.25	0.00162(4)	0.128(2)
c = 9.292270(8) Å	Br1	2*c*	0.66667	0.33333	0.75	0.00193(6)	1
V = 357.013(1) Å^3	F1	2*b*	1	0	0.75	0.00646(32)	1
$Cu_{3.38}Zn_{0.62}O_6FBr$	O1	12*k*	0.79853(7)	0.20147(7)	0.40625(9)	0.00084(16)	1
$\mathbf{Zn}_{0.56}{}^{d}$	Cu1	6*g*	0.5	0	0.5	0.00635(5)	1
$T = 295$ K	Zn1	2*d*	0.66667	0.33333	0.25	0.00911(36)	0.581(7)
a = 6.672118(14) Å	Cu2	6*h*	0.63010(119)	0.26014(237)	0.25	0.00635(5)	0.140(2)
c = 9.314972(4) Å	Br1	2*c*	0.66667	0.33333	0.75	0.01079(9)	1
V = 359.120(1) Å^3	F1	2*b*	1	0	0.75	0.01735(43)	1
$Cu_{3.42}Zn_{0.58}O_6FBr$	O1	12*k*	0.79808(8)	0.20192(8)	0.40627(10)	0.00388(19)	1

[a]Overall GOF = 2.82, Overall w*R* = 10.91%.
PXRD: w*R* = 12.99%. ZnOHF impurity: 0.01378(27) wt.%. NPD: w*R* = 4.71%.
[b]Overall GOF = 2.22, Overall w*R* = 8.94%.
PXRD: w*R* = 10.71%. ZnOHF impurity: 0.01460(26) wt.%. NPD: w*R* = 4.57%.
[c]GOF = 2.60, w*R* = 10.42%. LiF impurity: 0.01101(32 wt.%.
[d]GOF = 2.53, w*R* = 9.88%. LiF impurity: 0.00772(37) wt.%.

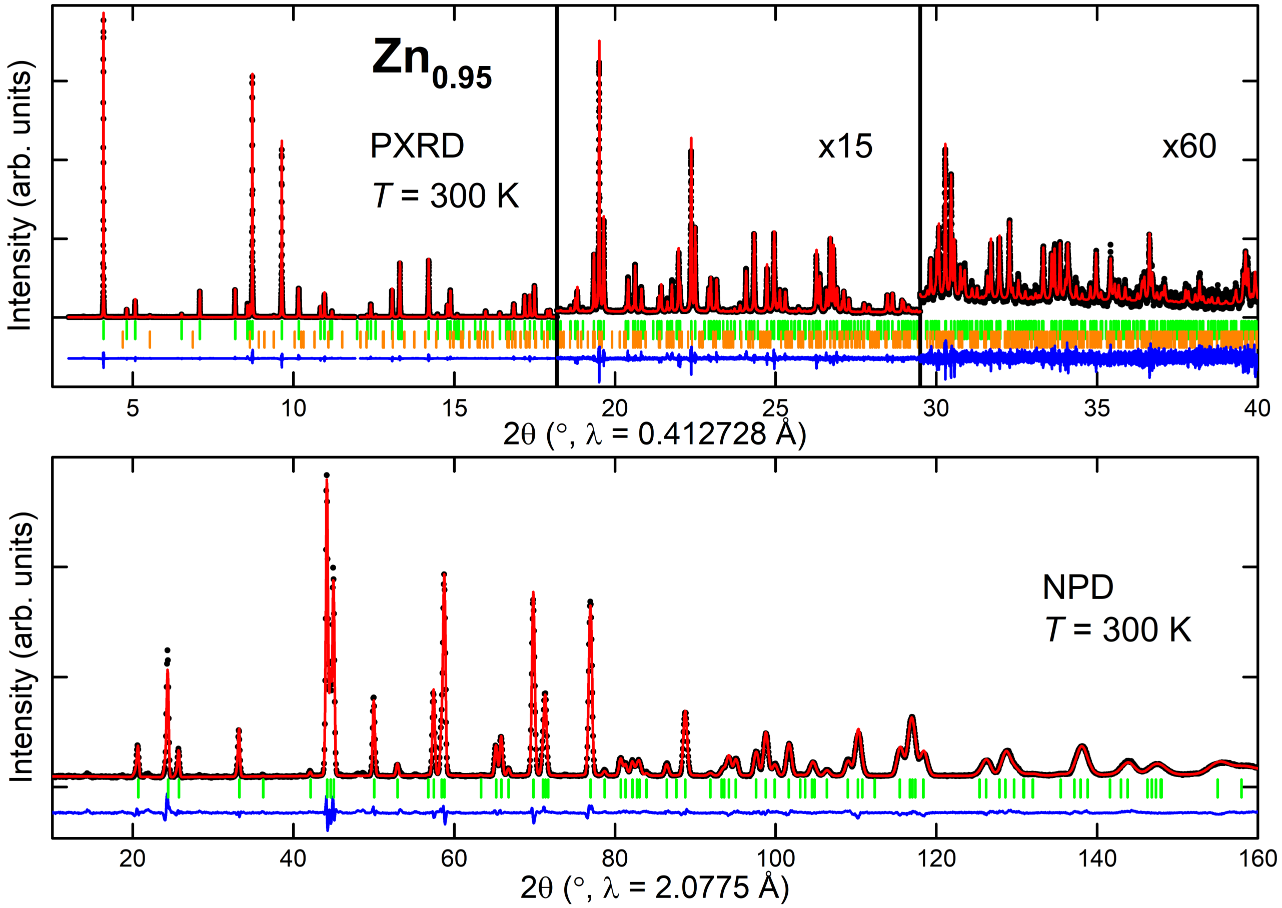

FIG. 8: Rietveld co-refinement of synchrotron PXRD (top) and NPD (bottom) data of deuterated $\mathbf{Zn}_{0.95}$ at $T = 300$ K. Observed (black), calculated (red), and difference (blue) plots are shown, and Bragg reflections are indicated by green tick marks. The Bragg reflections of a ZnOHF impurity phase are marked with orange tick marks.

TABLE 13: Selected bond angles relating to the kagome Cu's extracted from Rietveld refinements of Zn-substituted barlowite

**Radiation**<br>**Temperature**	$\mathbf{Zn}_{0.95}$<br>PXRD / NPD<br>13 / 2 K	$\mathbf{Zn}_{0.95}$<br>PXRD / NPD<br>300 K	$\mathbf{Zn}_{0.56}$<br>PXRD<br>90 K	$\mathbf{Zn}_{0.56}$<br>PXRD<br>295 K
Cu1–O1–Cu1	116.93(3)°	117.12(3)°	116.25(4)°	116.37(5)°
Cu1–O1–Zn1	95.868(9)°	95.965(9)°	95.750(10)°	95.94(3)°
Cu1–O1–Cu2 (1)			107.00(3)°	107.73(3)°
Cu1–O1–Cu2 (2)			92.12(17)°	92.17(18)°
Cu1–O1–Cu2 (3)			88.86(16)°	88.71(17)°

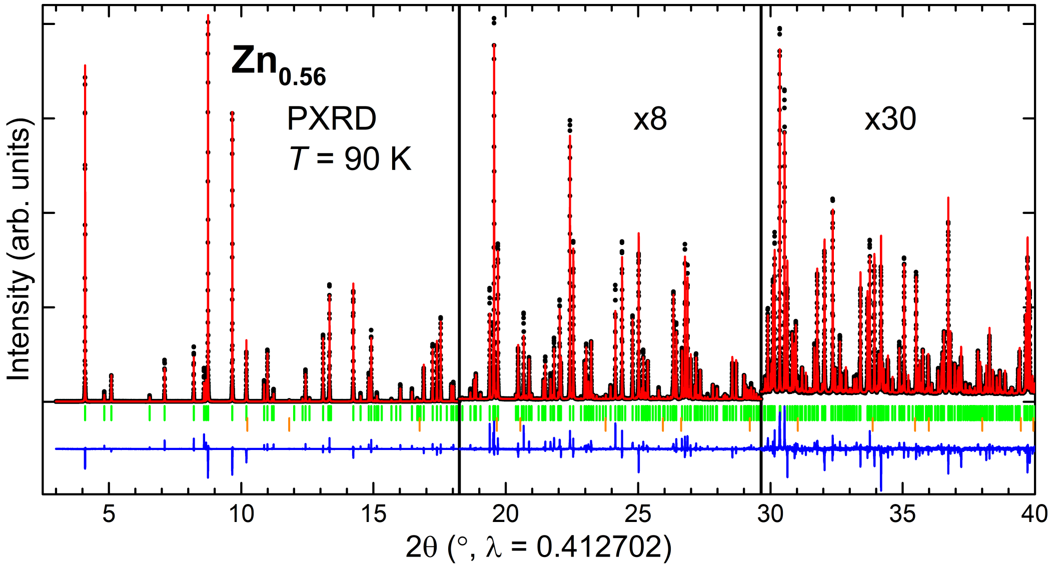

FIG. 9: Rietveld refinement of synchrotron PXRD data of protonated $\mathbf{Zn}_{0.56}$ at $T = 90$ K. Observed (black), calculated (red), and difference (blue) plots are shown, and Bragg reflections are indicated by green tick marks. The Bragg reflections of a LiF impurity phase are marked with orange tick marks.

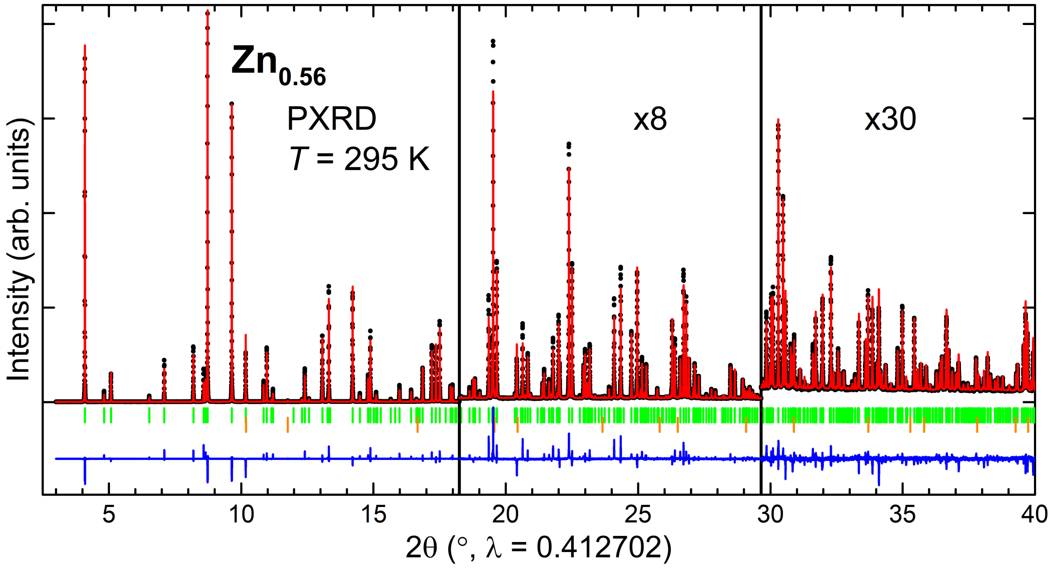

FIG. 10: Rietveld refinement of synchrotron PXRD data of protonated $\mathbf{Zn}_{0.56}$ at $T = 295$ K. Observed (black), calculated (red), and difference (blue) plots are shown, and Bragg reflections are indicated by green tick marks. The Bragg reflections of a LiF impurity phase are marked with orange tick marks.

TABLE 14: Selected bond distances in Å extracted from Rietveld refinements of **Zn**$_{0.95}$ and **Zn**$_{0.56}$

	**Zn**$_{0.95}$	**Zn**$_{0.95}$	**Zn**$_{0.56}$	**Zn**$_{0.56}$
**Radiation**	PXRD / NPD	PXRD / NPD	PXRD	PXRD
**Temperature**	13 / 2 K	300 K	90 K	295 K
Cu1–Cu1	3.33284(0)	3.33910(0)	3.33032(0)	3.33606(0)
Cu1–O1	1.9551(5)	1.9568(3)	1.9609(5)	1.9630(6)
Cu1–Br1	3.01714(0)	3.02373(0)	3.01557(0)	3.02205(0)
Zn1–O1	2.1067(6)	2.1108(6)	2.1029(7)	2.1036(8)
Cu1–Zn1	3.01714(0)	3.02373(0)	3.01557(0)	3.02205(0)
Cu2–O1 (1)			1.989(6)	1.988(6)
Cu2–O1 (2)			1.990(6)	1.987(6)
Cu2–O1 (3)			2.4261(6)	2.4417(7)
Cu2–Cu2			0.73167(3)	0.76496(3)
Cu1–Cu2 (1)			2.76544(0)	2.76162(0)
Cu1–Cu2 (2)			3.176(5)	3.191(5)
Cu1–Cu2 (3)			3.175(5)	3.190(5)

V. PHYSICAL PROPERTIES OF BARLOWITE AND ZN-SUBSTITUTED BARLOWITE

Curie-Weiss parameters are tabulated in Table 15 for bulk samples and Table 16 for aligned single crystalline **2** and $\mathbf{Zn}_{0.56}$. Aligned magnetization on a single crystal of barlowite **2** at $T = 2$ K and $T = 20$ K is shown in Figure 12. Figure 11 shows the aligned magnetization in the *ab* plane at $T = 2$–12 K; the curves below $T = 6$ K display a different slope than the curves where $T \geq 6$ K with a crossover at approximately $\mu_0 H = 0.75$–1 T. This change in slope coincides with the lower-temperature transition (T_{N2}) observed in AC susceptibility (Figure 4a in the main text) and supports the hypothesis that different spins are primarily responsible for each magnetic transition. DC susceptibility and inverse susceptibility measurements on bulk samples of barlowite **1**, barlowite **2**, Zn-substituted barlowite $\mathbf{Zn}_{0.95}$ and $\mathbf{Zn}_{0.56}$, and herbertsmithite at several applied magnetic fields are shown in Figures 13–14 An estimate of the susceptibility of the intrinsic barlowite kagome lattice is also plotted. Aligned single crystalline susceptibility and inverse susceptibility data of barlowite **2** and $\mathbf{Zn}_{0.56}$ are shown in Figure 15. Molar and magnetic heat capacity (C and C_{mag}, respectively) measurements on barlowite **2** are shown in Figure 16. Dilution refrigerator heat capacity measurements on bulk $\mathbf{Zn}_{0.95}$ and single crystalline $\mathbf{Zn}_{0.56}$ are shown in Figure 17. A comparison of the heat capacity of all samples is shown in Figure 18.

TABLE 15: Curie-Weiss parameters fit from $T = 150$–300 K for bulk samples (polycrystalline **1** and $\mathbf{Zn}_{0.95}$ and collections of single crystals of **2** and $\mathbf{Zn}_{0.56}$). All samples were measured in an applied field of $\mu_0 H = 7$ T, and no diamagnetic correction χ_0 was used. Note: 1 emu (mol Oe)$^{-1}$ = $4\pi 10^{-6}$ m^3 mol^{-1}

	**1**	**2**	$\mathbf{Zn}_{0.56}$	$\mathbf{Zn}_{0.95}$	Impurity-Subtracted $\mathbf{Zn}_{0.56}$
C (K emu (mol Cu)$^{-1}$)	0.384(2)	0.459(1)	0.443(2)	0.3951(3)	0.3914(3)
Θ (K)	-114(2)	-114(1)	-169(1)	-236(1)	-253(0)
μ_{eff} (μ_{B})	1.752(4)	1.911(3)	1.877(3)	1.778(1)	1.7695(7)
g-factor	2.023(5)	2.206(3)	2.167(4)	2.053(1)	2.0432(8)

TABLE 16: Curie-Weiss parameters fit from $T = 150$–300 K for single crystalline **2** and $\mathbf{Zn}_{0.56}$. All samples were measured in an applied field of $\mu_0 H = 7$ T, and no diamagnetic correction χ_0 was used. Note: 1 emu (mol Oe)$^{-1}$ = $4\pi 10^{-6}$ m^3 mol^{-1}

	Barlowite **2** *ab* plane	Barlowite **2** *c*-axis	Barlowite **2** Average	$\mathbf{Zn}_{0.56}$ *ab* plane	$\mathbf{Zn}_{0.56}$ *c*-axis	$\mathbf{Zn}_{0.56}$ Average
C (K emu (mol Cu)$^{-1}$)	0.453(2)	0.510(3)	0.471(2)	0.474(2)	0.547(2)	0.497(2)
Θ (K)	-110(1)	-145(2)	-121(1)	-160(1)	-195(1)	-171(1)
μ_{eff} (μ_{B})	1.904(3)	2.019(6)	1.941(4)	1.946(3)	2.091(4)	1.994(3)
g-factor	2.199(3)	2.331(7)	2.241(4)	2.248(3)	2.415(4)	2.303(3)

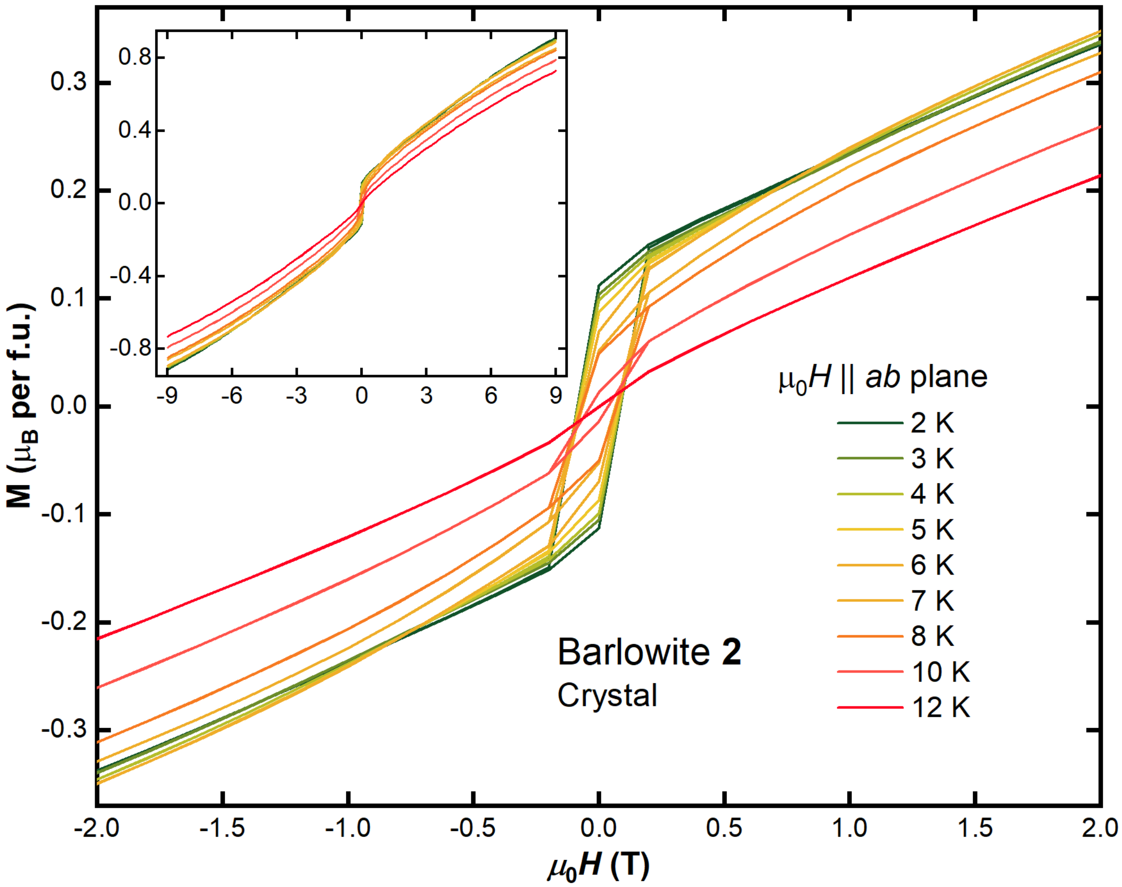

FIG. 11: Aligned magnetization as a function of applied field parallel to the *ab* plane from $T = 2$ K to $T = 12$ K for a single crystal of barlowite **2**. The inset shows the full range from $\mu_0 H = -9$–9 T. Note: 1 emu (mol Oe)$^{-1}$ = $4\pi 10^{-6}$ m^3 mol^{-1}.

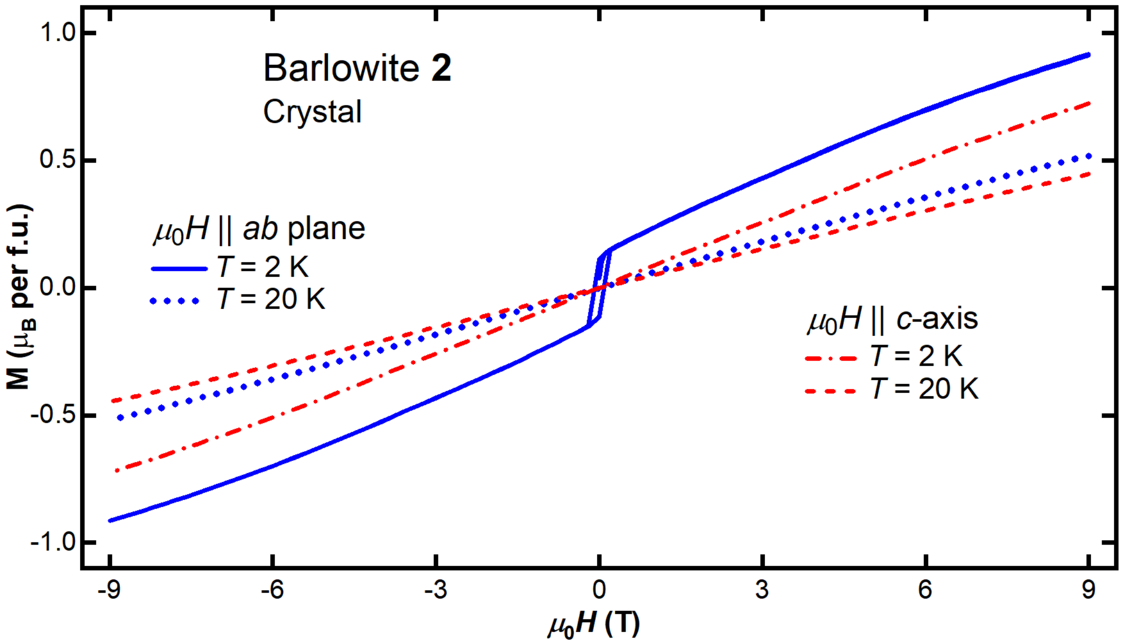

FIG. 12: Aligned magnetization as a function of applied field at $T = 2$ K and $T = 20$ K for a single crystal of barlowite **2**. Note: 1 emu (mol Oe)$^{-1}$ $= 4\pi 10^{-6}$ m^3 mol^{-1}.

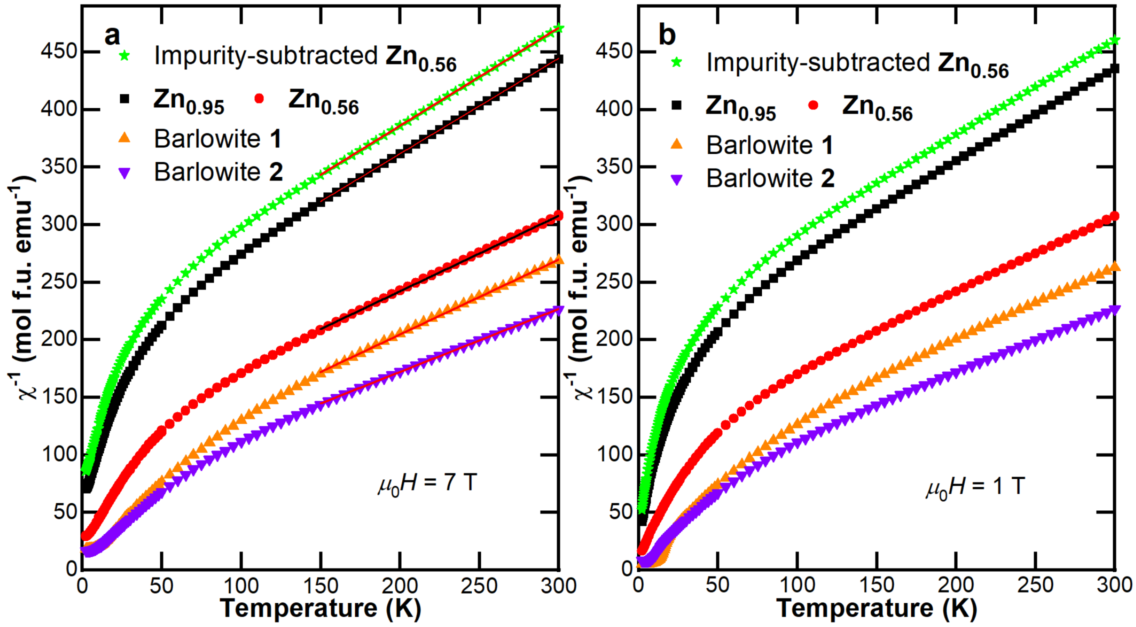

FIG. 13: Bulk inverse susceptibility data of barlowite **1**, barlowite **2**, **Zn**$_{0.95}$, **Zn**$_{0.56}$, and impurity-subtracted **Zn**$_{0.56}$ measured at **a** $\mu_0 H = 7$ T and **b** $\mu_0 H = 1$ T. The lines shown in **a** are Curie-Weiss fits from $T = 150$–300 K. Note: 1 emu (mol Oe)$^{-1}$ $= 4\pi 10^{-6}$ m^3 mol^{-1}.

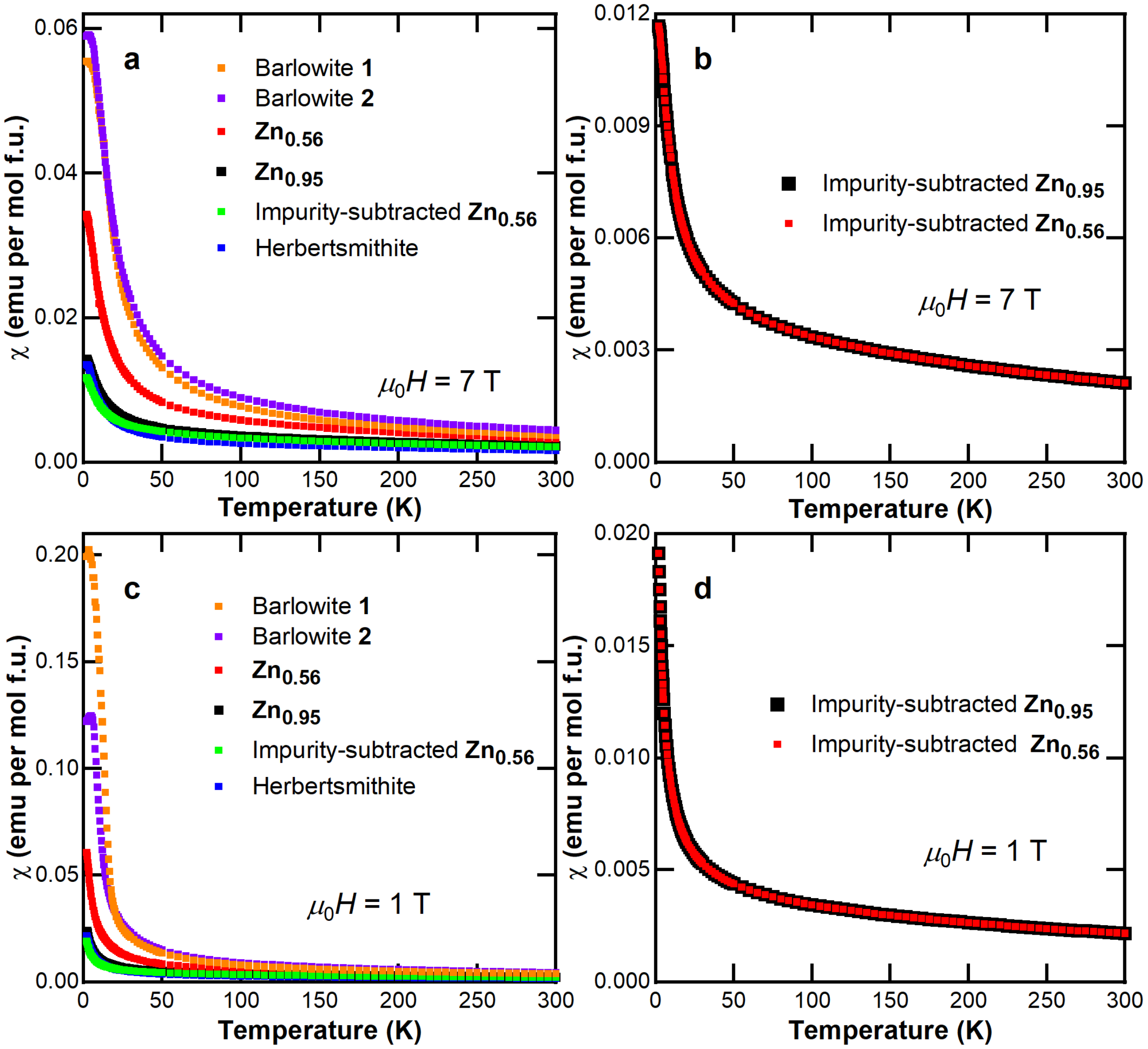

FIG. 14: Bulk susceptibility data of barlowite **1**, barlowite **2**, **Zn**$_{0.95}$, **Zn**$_{0.56}$, herbertsmithite, and impurity-subtracted **Zn**$_{0.56}$ measured at **a** $\mu_0 H = 7$ T and **c** $\mu_0 H = 1$ T. Impurity-subtracted susceptibility data for **Zn**$_{0.95}$ and **Zn**$_{0.56}$ measured at **b** $\mu_0 H = 7$ T and **d** $\mu_0 H = 1$ T. Note: 1 emu (mol Oe)$^{-1}$ $= 4\pi 10^{-6}$ m^3 mol^{-1}.

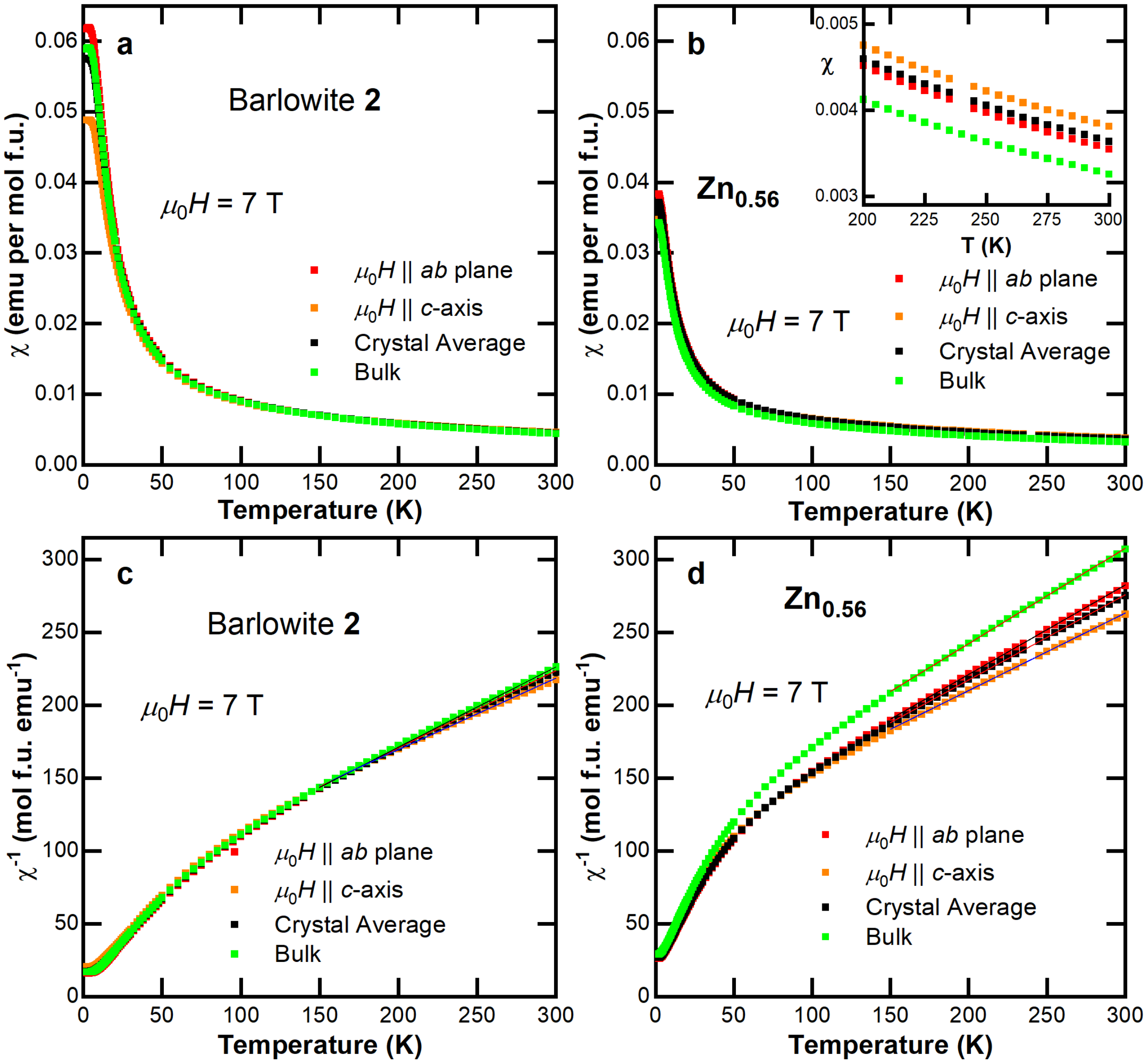

FIG. 15: Aligned susceptibility data of single crystalline **a** barlowite **2** and **b** $\mathbf{Zn}_{0.56}$ with the average $\chi_{\rm av} = (2/3\chi_{\rm ab} + 1/3\chi_{\rm c})$ compared to the respective bulk sample. The inset shows the high-temperature behavior. Aligned inverse susceptibility data of single crystalline barlowite **c** **2** and **d** $\mathbf{Zn}_{0.56}$ with the average $\chi_{\rm av}^{-1} = (2/3\chi_{\rm ab} + 1/3\chi_{\rm c})^{-1}$ compared to the respective bulk sample. The lines are Curie-Weiss fits from $T = 150$–300 K, and all data was measured at $\mu_0 H = 7$ T. Note: 1 emu (mol Oe)$^{-1}$ = $4\pi 10^{-6}$ m^3 mol^{-1}.

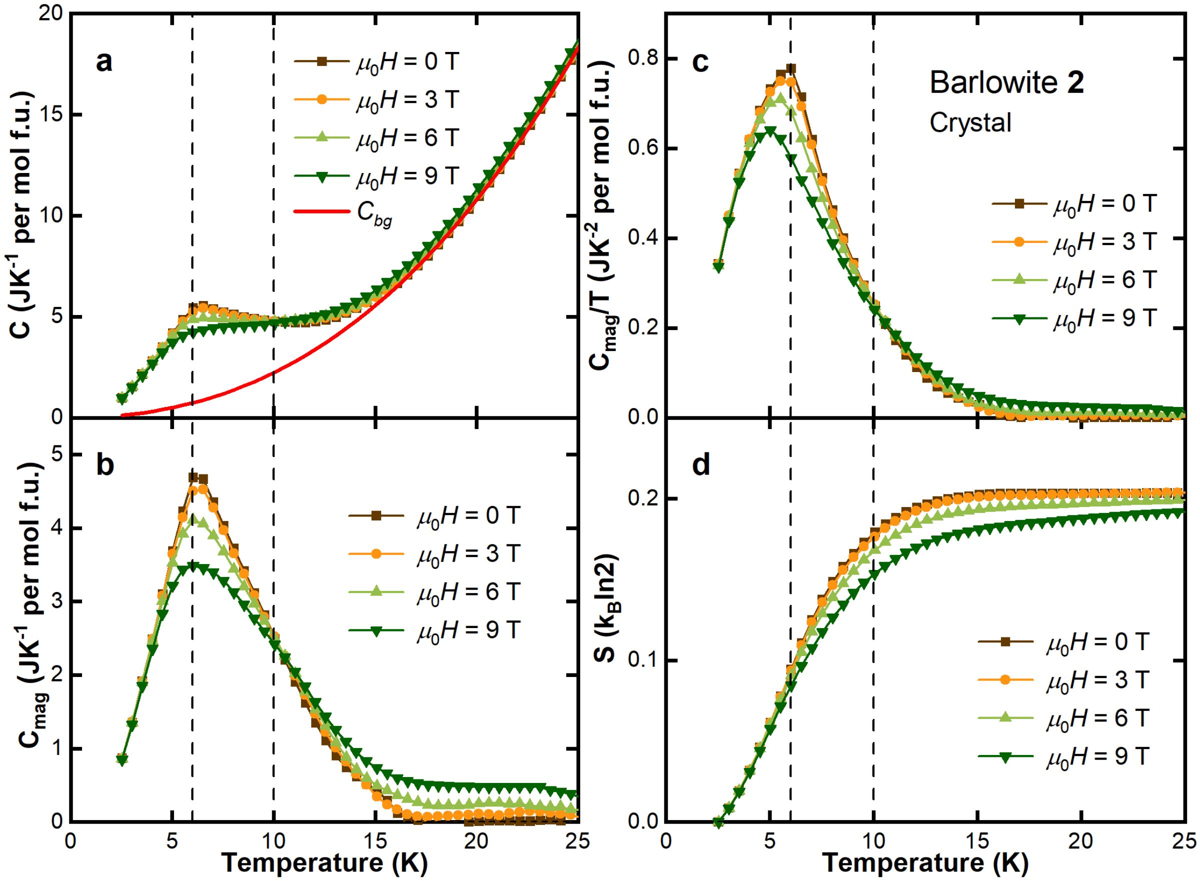

FIG. 16: **a** Molar heat capacity (C) of single crystalline barlowite **2** in applied fields up to $\mu_0 H = 9$ T. The red line is the background curve (C_{bg}) that was fit between $T = 20$–30 K. **b** Magnetic heat capacity (C_{mag}) calculated as $C - C_{\text{bg}}$. **c** Magnetic heat capacity divided by temperature ($C_{\text{mag}}T^{-1}$) of barlowite **2**. **d** Magnetic entropy (S) normalized as a fraction of the total value per Cu of a single crystal of barlowite **2**. The dashed black lines denote the magnetic transitions $T_{\text{N1}} = 10$ K and $T_{\text{N2}} = 6$ K.

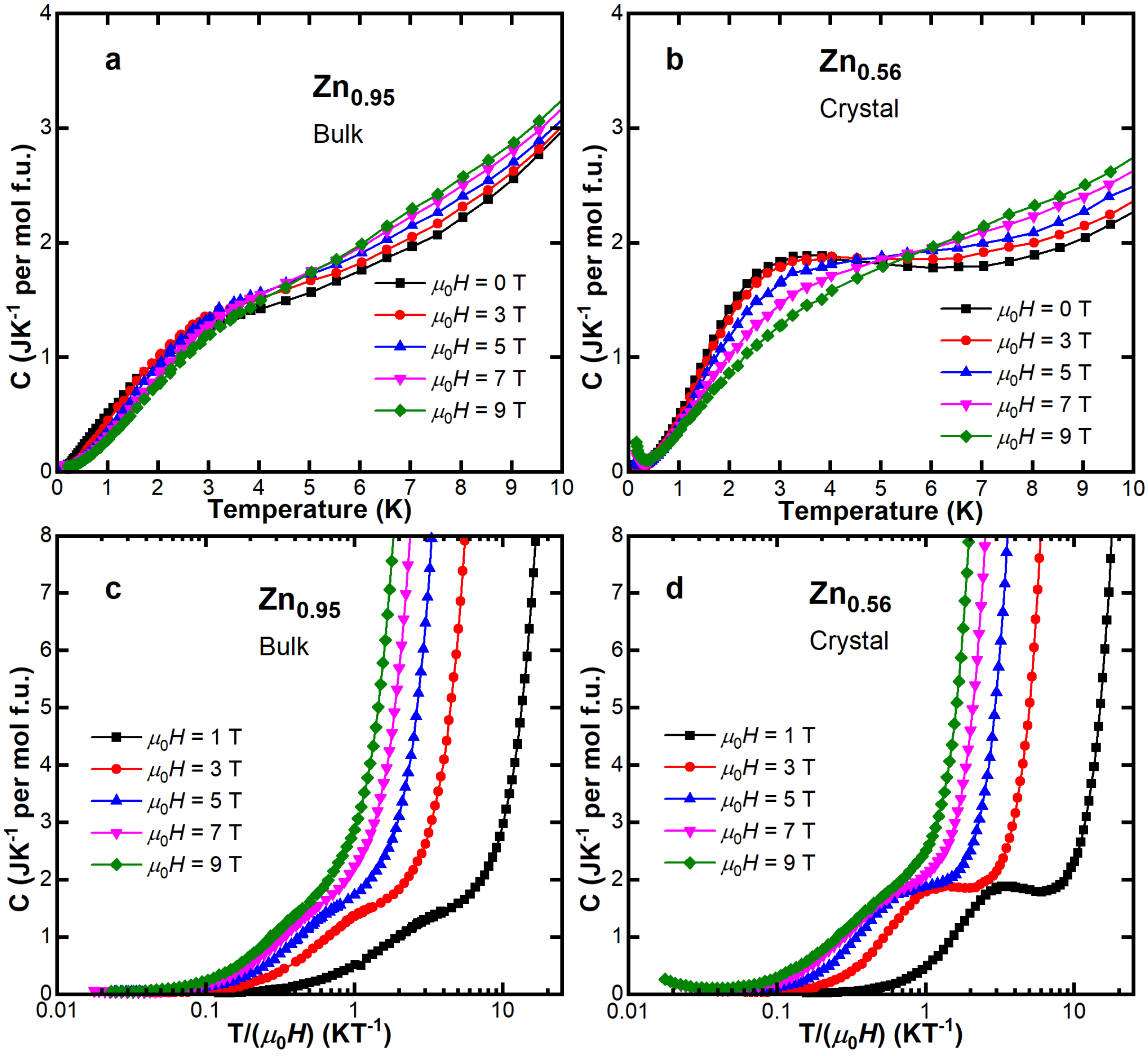

FIG. 17: Temperature dependence of molar heat capacity (C) for **a** bulk **Zn**$_{0.95}$ and **b** single crystalline **Zn**$_{0.56}$. Dependence of C upon $T(\mu_0 H)^{-1}$ for **c** **Zn**$_{0.95}$ and **d** **Zn**$_{0.56}$.

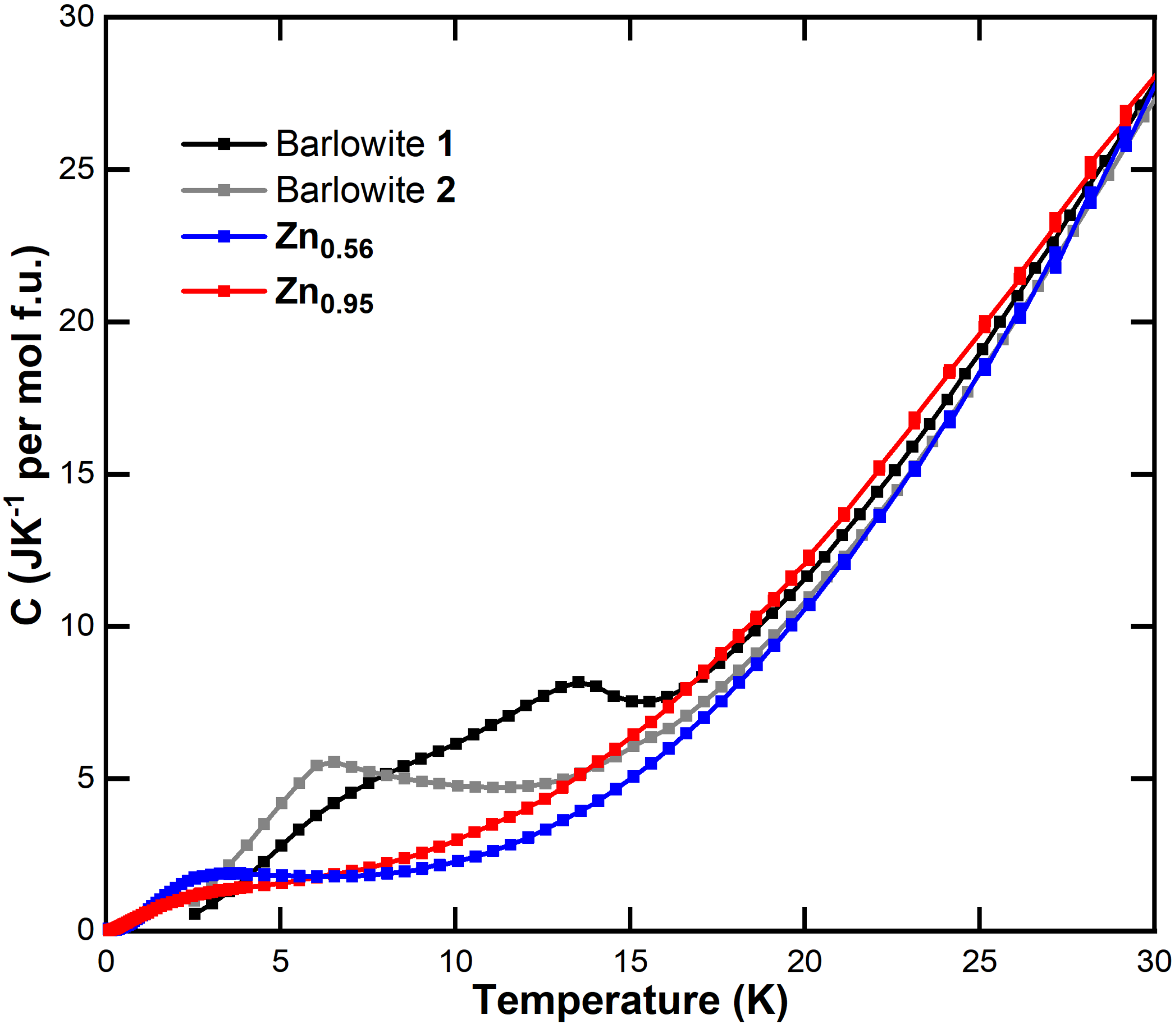

FIG. 18: Temperature dependence of molar heat capacity (C) for bulk barlowite **1** and $\mathbf{Zn}_{0.95}$ and single crystalline barlowite **2** and $\mathbf{Zn}_{0.56}$.

A. Magnetic measurements of deuterated barlowite 2

Deuterated crystals of barlowite **2** were synthesized for elastic neutron scattering measurements. Figure 19 shows the real part of the AC susceptibility (χ') and DC magnetization (M) as a function of temperature for one representative batch of crystals. The magnetic behavior is slightly different from that of protonated barlowite **2** (Figure 4a in the main text): the higher-temperature transition ($T_{\mathrm{N1}} = 10$ K) is more a shoulder instead of a distinct peak. The lower-temperature transition ($T_{\mathrm{N2}} = 6$ K) does not change significantly.

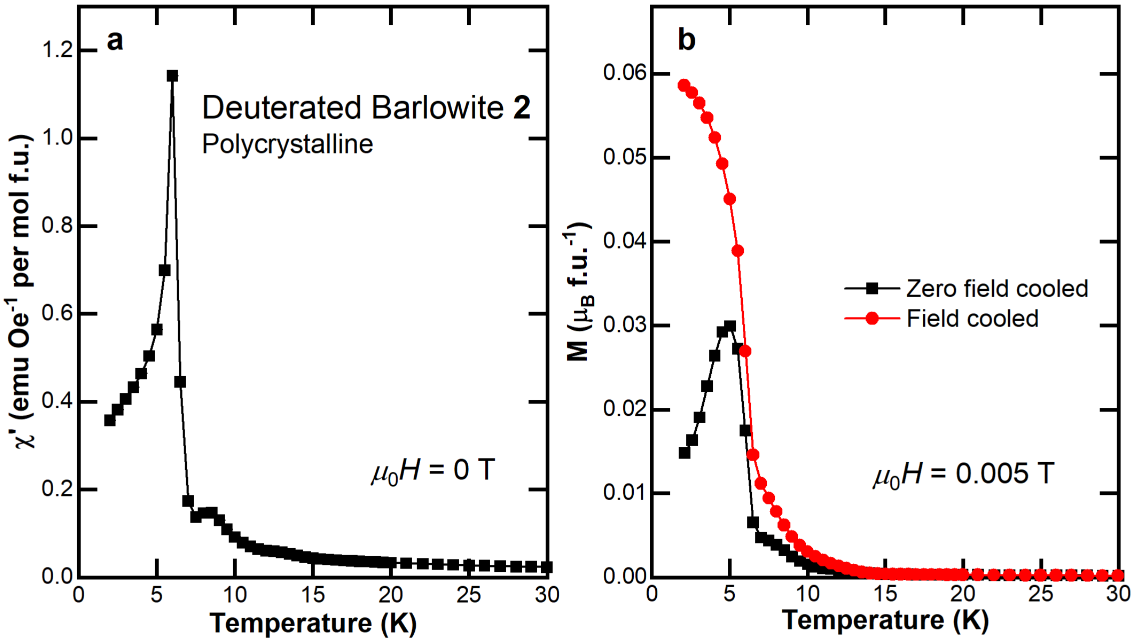

FIG. 19: Representative magnetic properties of bulk, polycrystalline deuterated barlowite **2**. **a** The real part of the AC susceptibility (χ') as a function of temperature measured at $\mu_0 H = 0$ T. **b** DC magnetization (M) as a function of temperature measured at $\mu_0 H = 0.005$ T. Note: 1 emu (mol Oe)$^{-1}$ $= 4\pi 10^{-6}$ m^3 mol^{-1}.

VI. ELASTIC NEUTRON SCATTERING OF BARLOWITE 2

A. Background subtraction and fitting methods for the superlattice peak

The notation of the high-temperature space group *P*6$_3$/*mmc* is used to index peaks. In reciprocal space, θ-2θ scans and θ scans are along the longitudinal and transverse directions, respectively, of the wavevector $\vec{Q}$ in the scattering plane. Figure 1d in the main text shows the temperature dependence of longitudinal (θ-2θ) scans of the [0.5 1.5 0] superlattice peak, which are fit well by a Gaussian peak with a sloping background. The background, peak center and peak width are fixed to the best overall value based on all temperatures. Figure 20a shows the temperature dependence of transverse (θ) scans of the [1.5 0.5 0] peak, which is an equivalent position of [0.5 1.5 0]. They are superlattice peaks that are allowed in *P*6$_3$/*m* but forbidden in *P*6$_3$/*mmc*. Transverse (θ) scans at base temperature (1.6 K) and the highest temperature measured (283 K) are shown in Figure 20b. Although there is still a bump in the $T = 283$ K data, its center position deviates significantly from the peak position expected from the lattice parameters, which is denoted by the vertical dashed line. Thus, the $T = 283$ K data is treated as a temperature independent background and is subtracted from the lower temperature scans. The $T = 1.6$ K scan has the best statistics and is fit well by a Gaussian peak. The peak center is fixed to the expected peak position, and the peak width is fixed to the best overall value based on all temperatures. The fitted integrated intensities from both data sets are shown in the inset of Figure 1d in the main text after scaling to match each other. The structural phase transition temperature derived from linear fits of both data sets is $T = 262(8)$ K.

B. Peak broadening of superlattice nuclear Bragg peaks and magnetic Bragg peaks

Neither the superlattice peaks nor the magnetic Bragg peaks at half-integer positions are resolution limited; however, the integer magnetic Bragg peaks are limited by the experimental resolution. Figure 21 shows representative scans of different types of magnetic Bragg peaks. Within a finite-size domain model,[42] the peak intensity is given by:

$$I \propto \frac{\sin^2 \frac{\Delta\vec{Q}}{2} \cdot N_1\vec{a}_1}{\sin^2 \frac{\Delta\vec{Q}}{2} \cdot \vec{a}_1} \frac{\sin^2 \frac{\Delta\vec{Q}}{2} \cdot N_2\vec{a}_2}{\sin^2 \frac{\Delta\vec{Q}}{2} \cdot \vec{a}_2} \frac{\sin^2 \frac{\Delta\vec{Q}}{2} \cdot N_3\vec{a}_3}{\sin^2 \frac{\Delta\vec{Q}}{2} \cdot \vec{a}_3}$$

These peaks are well-described by a Gaussian line shape, so we can make a Gaussian approximation of the above equation to match the peak maxima and integrated intensities. Assuming a cubic crystal ($a_1 = a_2 = a_3$) and isotropic crystallite ($N_1 = N_2 = N_3$), the domain size is related to the FWHM of the peak by:

$$L = \frac{4\sqrt{\pi ln2}}{\mathrm{FWHM}}$$

Therefore, we can extract the domain size from the fitted peak width. It is noted that the experimental resolution should be subtracted quadratically. The calculated domain sizes from

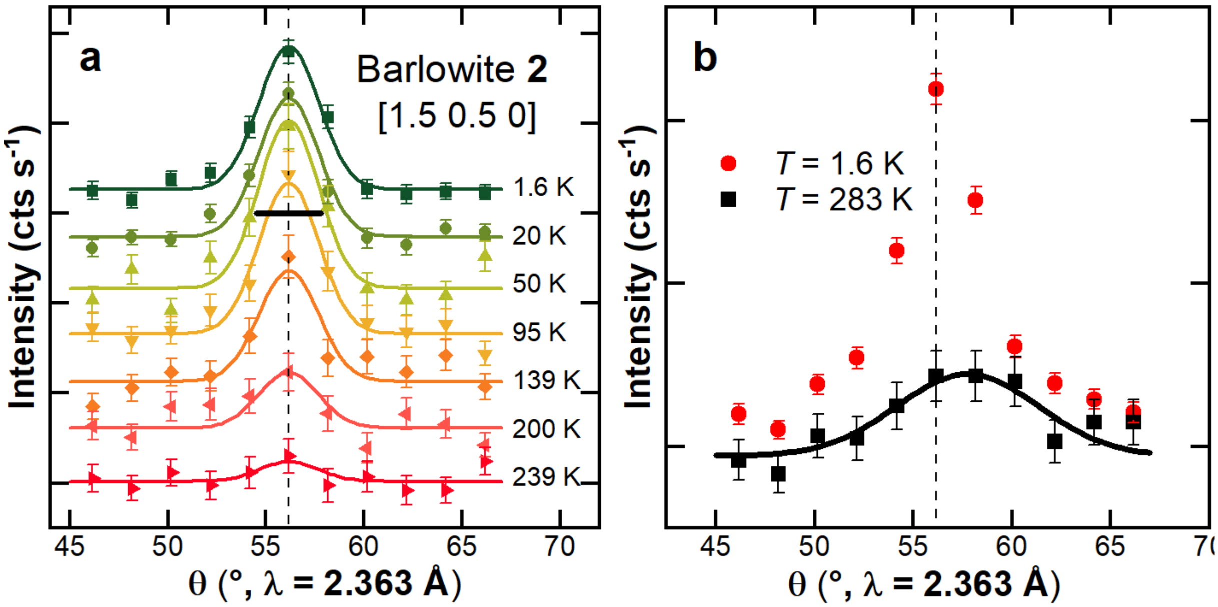

FIG. 20: **a** Elastic neutron scattering on single crystalline barlowite **2** showing the temperature dependence of the $P6_3/m$ superlattice peak [1.5 0.5 0] measured along the transverse direction of the wavevector $\vec{Q}$. The highest temperature data ($T = 283$ K) is subtracted as a background. **b** Transverse (θ) scans at base temperature ($T = 1.6$ K) and the highest temperature for the [1.5 0.5 0] superlattice peak. The vertical dashed lines represent the peak position expected from the lattice parameters. Scans were measured with $E_\mathrm{i} = 14.656$ meV at HB-1A. Uncertainties are statistical in origin and represent one standard deviation.

different scans at various peaks are listed in Table 17. For magnetic Bragg peaks, there are two coexisting distinct domain sizes: the half-integer peaks have a short domain size of $\sim$118 Å on average, while the integer peaks have a much larger domain size of at least $\sim$360 Å, which is limited by the experimental resolution.

C. Resolution function calculation and magnetic structure refinement method

For both nuclear and magnetic Bragg scattering, the neutron differential cross section can be written as:[69]

$$(\frac{d\sigma}{d\Omega})_{Bragg} = A\frac{F^2}{v^2} \tag{1}$$

where F is the static structure factor, v is the unit cell volume and A is a constant that depends on the sample geometry, incident flux, sample volume, and counting time. If these factors are all held fixed in a series of measurements, then A is simply an overall scale factor. For a material with a known crystal structure, one can determine the overall scale factor A by measuring nuclear Bragg peaks. Then a magnetic structure model can be fit to the measured magnetic Bragg peaks. However, the measured intensity is not simply the differential scattering

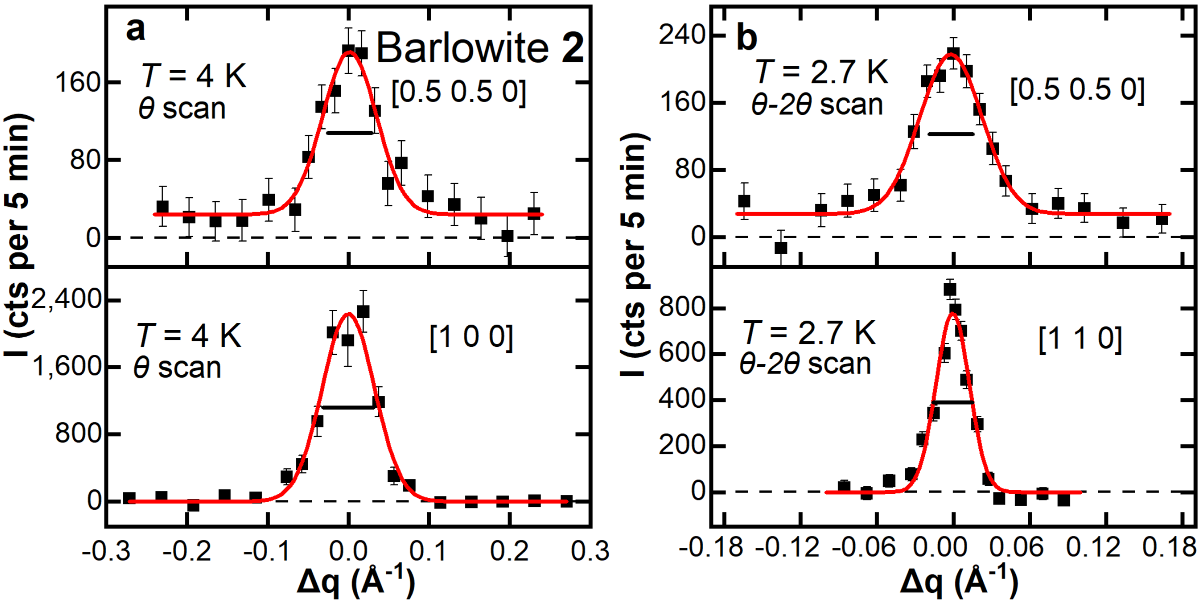

FIG. 21: **a** Transverse (θ) scans at the [0.5 0.5 0] and [1 0 0] magnetic Bragg peaks measured at $T = 4$ K. **b** Longitudinal (θ-2θ) scans at the [0.5 0.5 0] and [1 1 0] magnetic Bragg peak measured at $T = 2.7$ K. All measurements were taken with $E_\mathrm{i} = 5$ meV at SPINS with the highest temperature ($T = 20$ K) scan subtracted as a background.The solid lines are Gaussian fits, and the horizontal bars indicate the experimental resolution. Uncertainties are statistical in origin and represent one standard deviation.

cross section but rather a convolution of the differential cross section with the instrumental resolution:[69]

$$I(\vec{Q}_0) = \int \mathrm{d}\vec{Q} R(\vec{Q} - \vec{Q}_0) S(\vec{Q}) \tag{2}$$

where $S(\vec{Q}) = (\frac{d\sigma}{d\Omega})_{Bragg}$ is the differential cross section, and $R(\vec{Q} - \vec{Q}_0)$ is the resolution function. The resolution function depends on the specific spectrometer configuration, the sample mosaic spreads, the sample shape, and the wavevector $\vec{Q}$. In the Gaussian approximation, the resolution function can be expressed as a 3-dimensional (3D) Gaussian distribution:

$$R(\vec{Q} - \vec{Q}_0) = R_0 \exp(-\frac{1}{2} \Delta\vec{Q} M \Delta\vec{Q}) \tag{3}$$

where $M =$ is a 3×3 matrix and $\Delta\vec{Q} = \vec{Q} - \vec{Q}_0 = (q_x, q_y, q_z)$. The conventional coordinate system is the Q-system, in which x is along $\vec{Q}_0$, z is vertical, and y completes the orthogonal right-handed basis set. The resolution function can be visualized as a 3D ellipsoid centered at $\vec{Q}_0$. In the paraxial approximation, the resolution in the vertical direction is uncoupled from the other two in-plane coordinates. Hence the matrix elements $M_{xz} = M_{zx} = 0$ in M. For a

TABLE 17: Calculated domain sizes of barlowite **2** from various peaks for various scan types. For the superlattice peaks and the half-integer magnetic peaks, the mean domain size is calculated as a weighted mean. The mean domain size for the integer peaks is selected as the maximum value

	**Scattering plane**	**Instrument**	**Q**	**Scan type**	**Domain size (Å)**	**Mean domain size (Å)**
**Superlattice peak**	(H K 0)	HB-1A	[1.5 0.5 0]	Transverse	77(20)	61(4)
	(H K 0)	HB-1A	[1.5 0.5 0]	Longitudinal	97(22)	
	(H K 0)	SPINS	[0.5 1.5 0]	Longitudinal	36(5)	
	(H K 0)	SPINS	[0.5 1.5 0]	Longitudinal	94(6)	
**Magnetic peak**	(H K 0)	HB-1A	[0.5 0.5 0]	Transverse	90(25)	118(9)
	(H K 0)	SPINS	[0.5 0.5 0]	Transverse	105(17)	
	(H K 0)	SPINS	[0.5 0.5 0]	Longitudinal	127(13)	
	(H H L)	BT-7	[0.5 0.5 0]	Transverse	149(30)	
	(H K 0)	SPINS	[1 0 0]	Transverse	158(37)	360(107)
	(H K 0)	SPINS	[1 1 0]	Longitudinal	360(107)	
	(H K 0)	BT-7	[1 0 0]	Transverse	231(106)	
	(H 0 L)	BT-7	[1 0 0]	Transverse	300(182)	
	(H H L)	BT-7	[1 1 0]	Transverse	202(76)	
	(H H L)	BT-7	[1 1 0]	Longitudinal	220(35)	
	(H H L)	BT-7	[1 1 1]	Transverse	267(145)	

nuclear Bragg peak, assuming a perfect crystal, we can write:

$$S(\vec{Q}) = S_0\delta(\vec{Q} - \vec{Q}_b) \tag{4}$$

where S_0 is the amplitude of the scattering cross section at the Bragg peak $\vec{Q}_b$. Then the measured intensity is just the product of S_0 and the resolution function $R(\vec{Q}_b - \vec{Q}_0)$. For a scan through a Bragg peak $\vec{Q}_b$, the integrated intensity is given by:

$$\text{Int.}I(\vec{Q}_b) = S_0 \int R(\vec{Q}_b - \vec{Q}_0)\mathrm{d}\vec{Q}_0 \tag{5}$$

Thus, the integrated intensity not only depends on the differential cross section at the Bragg condition but also on the integral of the resolution function along the path of the scan in the reciprocal space.

Note that Eqn. 5 can only apply to a perfect crystal. For short-range ordered structures such as the magnetic order in barlowite **2**, the delta function approximation (Eqn. 4) no longer holds. However, we can still use Eqn. 5 with a correction factor that is based on three simple assumptions. First, the cross section $S(\vec{Q})$ can be modeled by a 3D Gaussian function centered at the Bragg peak $\vec{Q}_b$:

$$S(\vec{Q}) = S_0 \frac{1}{\sqrt{2\pi\sigma_x^2}} \exp(-\frac{q_x^2}{2\sigma_x^2}) \frac{1}{\sqrt{2\pi\sigma_y^2}} \exp(-\frac{q_y^2}{2\sigma_y^2}) \frac{1}{\sqrt{2\pi\sigma_z^2}} \exp(-\frac{q_z^2}{2\sigma_z^2}) \quad (6)$$

Second, the resolution function $R(\vec{Q}-\vec{Q}_0)$ can be approximated by $R(\vec{Q}-\vec{Q}_b)$, which—similar to $S(\vec{Q})$—is peaked at $\vec{Q}_b$. We assume that the resolution function varies slowly in the small region of a scan. Third, the off-diagonal elements $M_{xy} = M_{yx} \doteq 0$ in the matrix M because the major axis of the resolution ellipsoid is approximately aligned with the y-axis due to the large sample mosaic. Since the convolution of two Gaussian functions is also a Gaussian, we can obtain a formula for integrated intensity similar to Eqn. 5 but with a correction factor that depends on the scan direction. For example, the correction factor for θ scan in the $(H\ K\ 0)$ scattering plane is $1/\sqrt{(M_{xx}\sigma_x^2+1)(M_{zz}\sigma_z^2+1)}$.

The resolution function calculation in this work was performed using the Python module NeutronPy. The nuclear structure factor calculation was performed using VESTA[74] with input crystal structure data from the SCXRD refinement results (Table 3). Since the SCXRD cannot unambiguously determine the positions of H (or D) atoms due to their negligible X-ray scattering lengths, the overall scale factor A was calculated by measuring the nuclear Bragg peak [0 0 2], whose intensity is negligibly affected by these atoms. The magnetic structure refinement was accomplished through a least squares minimization routine written in Python to fit the calculated magnetic Bragg peak intensities to the measured integrated intensities. For simplicity, the Debye-Waller factor was neglected; the Landé splitting factor was fixed to 2; and an isotropic Cu^{2+} magnetic form factor was used. The measured intensities at equivalent positions were averaged to increase statistics. The small differences in base temperatures between different measurements was neglected based on the fact that the intensities saturate at $T \sim 3$ K (see Figure 5b in the main text). The two magnetic peaks at integer positions ([1 0 0] and [1 1 0]) measured at SPINS were excluded from the refinement since these two peaks were measured at $T = 4$ K, a temperature at which the intensities are not saturated. The peak broadening correction factors for all half-integer peaks used the same mean domain size of 118 Å, while the integer peaks used the same domain size of 360 Å, obtained from Table 17.

D. Detailed refinement results of the magnetic models

Possible magnetic space groups were generated using the k-Subgroupsmag application on the Bilbao Crystallographic Server.[70–73] As shown in Table 18, the highest-symmetry magnetic space group allowing a net in-plane moment (necessitated by the measured bulk magneti-

TABLE 18: Possible magnetic space groups and their allowed net magnetic moment direction for the $\vec{k}$ =(0,0,0) propagation vector in structural space group $P6_3/m$

**Magnetic space group**	**Net magnetic moment direction**
$P\bar{6}$	Along the *c*-direction
$P\bar{6}'$	No net moment
$P6_3$	Along the *c*-direction
$P6_3'$	No net moment
$P\bar{3}$	Along the *c*-direction
$P\bar{3}'$	No net moment
$P2_1/m$	Along the *c*-direction
$P2_1/m'$	No net moment
$P2_1'/m$	No net moment
$P2_1'/m'$	Within the *ab*-plane

zation in Figure 4c in the main text as well as Figures 11–12) is $P2_1'/m'$ with lattice parameters $2a \times 2b \times c$, which is the same as the low-temperature nuclear unit cell. Its symmetry operations reduce the number of independent Cu^{2+} sites in a unit cell to 10 (six kagome and four interlayer sites) and constrain the interlayer Cu^{2+} moments to be within the *ab* plane. Since we measured a limited number of magnetic peaks (17 distinct peaks in total and 12 peaks after taking equivalent positions into account), we further reduce the number of fitted sites to six (four interlayer and two kagome).

Table 19 shows the refinement results for the pinwheel *q=0* kagome model and the orthorhombic model, which is based upon previous models for polycrystalline, orthorhombic samples similar to barlowite **1**[34, 35] and is tested for completeness. Table 21 gives the measured and calculated intensities for each model. Both models give equally good fits, and we cannot unambiguously determine which is the best model from our data. However, the orthorhombic structure is less likely to exist in barlowite **2**, which has less distorted kagome planes and should therefore prefer a *q=0* type spin structure. Additionally, the orthorhombic model is not compatible with the underlying crystal structure, since the two types of kagome spins do not correspond to the two crystallographically distinct sites.

In the pinwheel *q=0* kagome model discussed in the main text, we used 10 fitting parameters. There are two types of kagome spins where each has a distinct moment size and in-plane rotation angle, but they share an out-of-plane rotation angle. There is one interlayer spin moment size and four different in-plane rotation angles of the interlayer spins. Figure 22a shows the moment directions when the rotation angles are at zero. Counterclockwise is the positive direction for all in-plane rotation angles. The direction of the out-of-plane rotation for each kagome spin is illustrated in Figure 5c in the main text and Figure 23a. Since the number of

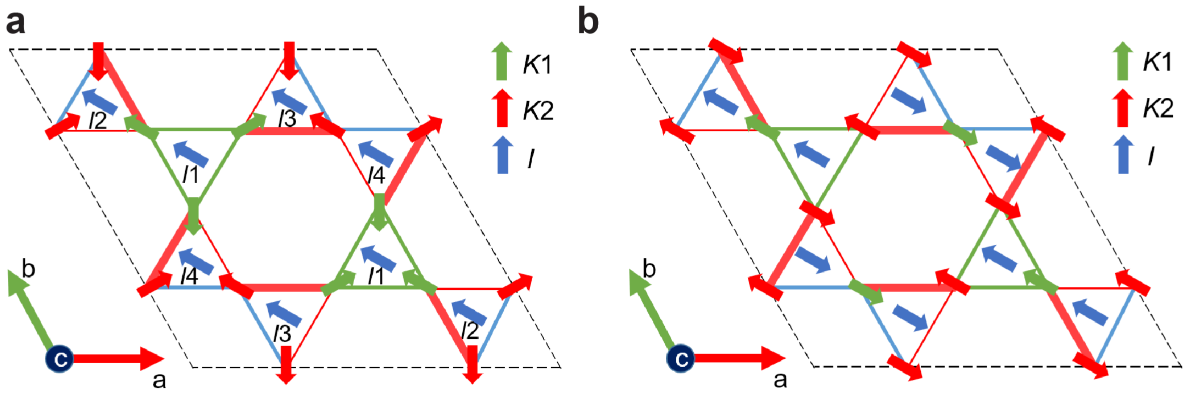

FIG. 22: Schematics of the spin structures of **a** the pinwheel *q=0* kagome model and **b** the orthorhombic model. All angles fit for the moment directions are fixed at zero, and the two types of kagome moments are denoted $K1$ and $K2$. Each interlayer motif is visualized by one spin at the weighted center of the three partially-occupied sites. The thickness of the lines between kagome spins indicates relative bond strength extracted from the Cu–O–Cu bond angles. The dashed lines denote the magnetic unit cell.

fitting parameters (10) is close to the number of distinct peaks (12), and the three unequal-occupancy interlayer sites are along the [1 1 0]-type high-symmetry directions within error, we fix those spin directions in the final fit (Table 19). This choice of spin directions for these three interlayer sites leads to zero net moment from them, which reduces the overall net moment size. However, we cannot exclude the possibility that these interlayer spins deviate from the specified directions and contribute to the net magnetization.

In addition, the possibility that the kagome plane has net magnetization is also tested by introducing an in-plane canting angle for the green kagome spins that have an out-of-plane component. The positive direction for this canting angle is towards the direction of the green kagome spins that lie within the kagome plane. To reduce the number of fitting parameters, the moment sizes are fixed. The fit result (Table 20) shows a small but non-zero canting angle ($-7°$) with a large error bar (20°) and a tiny change to the χ^2. No other rotation angles are significantly affected. Although this canting direction is opposite to the net moment direction from the interlayer spins, the χ^2 only worsens a small amount (from 39.5 to 40.2) if the canting angle is fixed to a positive value (Table 20). This positive canting of kagome moments contributes a net moment of 0.016 μ_B per formula unit.

In the orthorhombic model, we used seven fitting parameters. There are two types of kagome spins where each has a distinct moment size and in-plane rotation angle, but they share one out-of-plane canting angle. There is one interlayer spin moment size and one in-plane canting angle of the interlayer spins. Figure 22b shows the moment directions when the canting angles are fixed at zero. The positive direction for all the in-plane canting angles is towards the [1 1 0] direction. The direction of the out-of-plane canting for each kagome spin is illustrated in Figure 23b. Since our data is insensitive to the two in-plane canting angles of the kagome spins, we fix these angles to be zero in the final fit (Table 19).

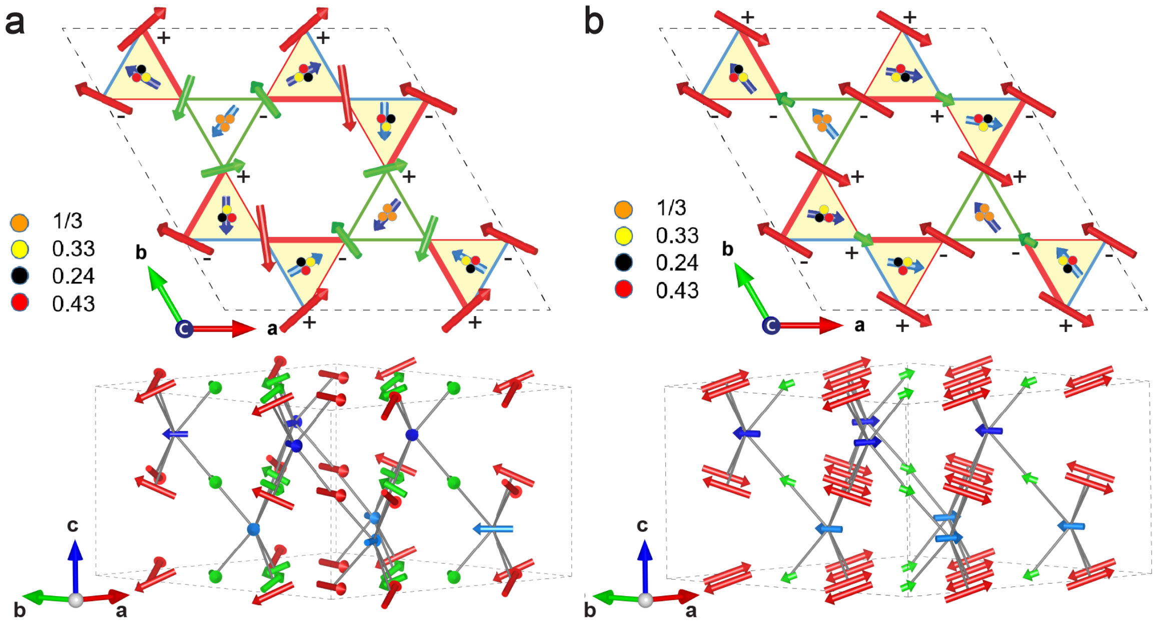

FIG. 23: **a** The pinwheel *q=0* kagome magnetic model and **b** the orthorhombic magnetic model of barlowite **2** in the *ab* plane (top) and an isometric view (bottom), visualized in VESTA.[74] The arrows indicate the sizes and directions of the moments. Dark and light blue spins represent interlayer Cu^{2+} ions in different layers, while red and green spins represent kagome Cu^{2+} ions. The three partially-occupied interlayer sites are shown with different colors, but each interlayer motif is visualized by one spin at the weighted center of the sites. The thickness of the lines between kagome spins indicates relative bond strength extracted from the Cu–O–Cu bond angles. The symbols next to the kagome spins denote the directions of the out-of-plane component in the top layer. The dashed lines denote the magnetic unit cell.

Both models have a smaller average ordered kagome moment compared to the proposed models for orthorhombic barlowite. It is markedly reduced from 0.32 μ_B per Cu (Ref. [35]) to 0.19 μ_B per Cu in both models. This is supported by the considerably weaker magnetic peaks of barlowite **2**: the ratio between the magnetic and nuclear contributions to the [1 0 0] Bragg peak is ∼1/90, which is ∼13 times smaller than for orthorhombic barlowite **1**.[34]

Tables 22 and 25 compare the kagome moments' and interlayer moments' contributions at various magnetic Bragg peaks for the pinwheel *q=0* kagome model and the orthorhombic model.

TABLE 19: Refinement results of the two magnetic models. The values marked with asterisks are fixed. For each model, the initial fit is a global refinement with all fitting parameters refined, while some parameters to which the model is insensitive are fixed in the final fit

			**Pinwheel *q=0* kagome model**		**Orthorhombic model**	
			Initial	Final	Initial	Final
**Moment size** (μ_B)	$K1$		0.15(4)	0.16(3)	0.08(2)	0.08(1)
	$K2$		0.24(4)	0.23(2)	0.24(3)	0.24(1)
	I		0.13(10)	0.13(2)	0.14(2)	0.14(1)
**Rotation/Canting angle** (°)	in-plane	$K1$	99(9)	100(4)	0.17(90)	0*
		$K2$	127(48)	128(5)	-0.04(90)	0*
		$I1$	67(179)	81(176)	20(49)	20(7)
		$I2$	-12(90)	0*		
		$I3$	294(179)	240*		
		$I4$	131(134)	120*		
	out-of-plane	$K1$	29(2)	29(2)	20(3)	20(1)
		$K2$				
χ^2			35.0	39.8	34.2	34.2
**Net moment** (μ_B **per formula unit**)			0.041	0.033	0.049	0.049

TABLE 20: Effect of canting the $K1$ kagome moments upon the pinwheel *q=0* kagome model. The values marked with asterisks are fixed. If the canting direction is not constrained, it aligns towards the negative direction. However, the fit is not significantly affected when the canting angle is fixed to the positive direction

<table>
<tr><th colspan="3" rowspan="2"></th><th rowspan="2">Without canting</th><th colspan="2">With canting</th></tr>
<tr><th>Negative in-plane canting</th><th>Positive in-plane canting</th></tr>
<tr><td rowspan="3">Moment size (μ_B)</td><td colspan="2">$K1$</td><td>0.16(3)</td><td>0.16*</td><td>0.16*</td></tr>
<tr><td colspan="2">$K2$</td><td>0.23(2)</td><td>0.23*</td><td>0.23*</td></tr>
<tr><td colspan="2">I</td><td>0.13(2)</td><td>0.13*</td><td>0.13*</td></tr>
<tr><td rowspan="9">Rotation/Canting angle (°)</td><td>in-plane canting</td><td>$K1$</td><td>0*</td><td>-7(21)</td><td>7*</td></tr>
<tr><td rowspan="6">in-plane rotation</td><td>$K1$</td><td>100(4)</td><td>100(6)</td><td>101(2)</td></tr>
<tr><td>$K2$</td><td>128(5)</td><td>129(2)</td><td>128(2)</td></tr>
<tr><td>$I1$</td><td>81(176)</td><td>93(172)</td><td>59(151)</td></tr>
<tr><td>$I2$</td><td>0*</td><td>0*</td><td>0*</td></tr>
<tr><td>$I3$</td><td>240*</td><td>240*</td><td>240*</td></tr>
<tr><td>$I4$</td><td>120*</td><td>120*</td><td>120*</td></tr>
<tr><td rowspan="2">out-of-plane rotation</td><td>$K1$</td><td rowspan="2">29(2)</td><td rowspan="2">29(1)</td><td rowspan="2">29(1)</td></tr>
<tr><td>$K2$</td></tr>
<tr><td colspan="3">χ^2</td><td>39.8</td><td>39.5</td><td>40.2</td></tr>
<tr><td colspan="3">Net moment (μ_B per formula unit)</td><td>0.033</td><td>0.019</td><td>0.046</td></tr>
</table>

TABLE 21: Measured and calculated integrated intensities of magnetic Bragg peaks from θ scans for the two magnetic models

**Instrument**	**Scattering plane**	**Q**	I_{calc} **(cts per** 5 **min)**		I_{exp} **(cts per** 5 **min)**
			Pinwheel *q=0* kagome model	Orthorhombic model	
HB-1A	(H K 0)	[0.5 0.5 0]	577	581	549(47)
		[1.5 1.5 0]	121	116	92(50)
	(H 0 L)	[0.5 0 1]	189	249	274(82)
		[1.5 0 2]	236	224	180(26)
		[0.5 0 2]	37	2	238(99)
		[0.5 0 3]	183	177	150(109)
SPINS	(H K 0)	[0.5 0.5 0]	920	926	816(51)
BT-7	(H K 0)	[0.5 0.5 0]	2077	2090	2207(334)
		[1 0 0]	24870	24812	23668(1512)
		[1 1 0]	5519	5522	5859(445)
	(H 0 L)	[0.5 0 1]	525	690	1095(209)
		[0.5 0 3]	720	693	577(115)
		[1 0 0]	33633	33555	35777(2191)
	(H H L)	[1 1 1]	3352	3359	3345(181)
		[1 1 0]	6075	6078	5892(338)
		[0.5 0.5 0]	2256	2270	2716(132)
		[0.5 0.5 2]	158	124	203(150)
		[0.5 0.5 1]	14	27	242(417)
		[0.5 0.5 3]	7	11	220(223)

TABLE 22: Contributions to the calculated intensities of magnetic Bragg peaks from different Cu^{2+} sites in the pinwheel $q = 0$ kagome model

**Instrument**	**Scattering plane**	**Q**	I_{calc} **(cts per** 5 **min)**		
			All	Only kagome	Only interlayer
HB-1A	(H K 0)	[0.5 0.5 0]	577	116	184
		[1.5 1.5 0]	121	34	28
	(H 0 L)	[0.5 0 1]	189	4	183
		[1.5 0 2]	236	52	31
		[0.5 0 2]	37	140	67
		[0.5 0 3]	183	4	150
SPINS	(H K 0)	[0.5 0.5 0]	920	186	293
BT-7	(H K 0)	[0.5 0.5 0]	2077	419	662
		[1 0 0]	24870	24382	33
		[1 1 0]	5519	5571	81
	(H 0 L)	[0.5 0 1]	525	11	508
		[0.5 0 3]	720	16	587
		[1 0 0]	33633	32974	44
	(H H L)	[1 1 1]	3352	3344	8
		[1 1 0]	6075	6132	89
		[0.5 0.5 0]	2256	455	719
		[0.5 0.5 2]	158	470	787
		[0.5 0.5 1]	14	14	0.2
		[0.5 0.5 3]	7	6	0.1

E. The orthorhombic model

Like in the pinwheel *q=0* model, in the orthorhombic model the kagome spins (the red and green spins in Figure 22b) have two moment sizes (0.08(1) μ_B and 0.24(1) μ_B). However, this classification is not consistent with the two crystallographically distinct sites, which are denoted Cu1 and Cu2 in the data for barlowite **2** in Tables 3 and 9. The out-of-plane canting of the kagome moments also forms alternating stacking between layers, giving rise to the [1 1 1] magnetic peak. It is necessary to have two distinct kagome sites and cant the interlayer site: it matches the measured peak intensity of the [0.5 0 1] and [0.5 0 3] peaks better than using one kagome site does, and including the canting angle of interlayer Cu^{2+} as a refined parameter significantly improves the fit, especially for the integer peaks (Tables 23–24). The interlayer spins have a moment size of 0.14(1) μ_B. The in-plane canting of the interlayer spins also results in net FM with a net moment of 0.05 μ_B per formula unit. The fit becomes much worse if the net moment size is constrained to 0.11 μ_B per formula unit (see Tables 23–24).

TABLE 23: Comparisons of refinement results of the orthorhombic model with different constraints. The values marked with asterisks are fixed

			**No constraints**	**One type of kagome**	**No interlayer canting**	**Net moment constraint**
**Moment size** (μ_B)	*K*1		0.08(1)	0.19(1)	0.08(2)	0.08(2)
	*K*2		0.24(1)		0.22(1)	0.27(1)
	I		0.14(1)	0.07(3)	0.13(1)	0.17(1)
**Canting angle** (°)	in-plane	*K*1	0*	0*	0*	0*
		*K*2				
		I	20(7)	46(15)	0*	40(1)
	out-of-plane	*K*1	20(1)	26(3)	22(1)	18(1)
		*K*2				
χ^2			34.2	175.2	52.4	63.3
**Net moment (**μ_B **per formula unit)**			0.049	0.048	0	0.110

TABLE 24: Measured and calculated integrated intensities of magnetic Bragg peaks from θ scans for the orthorhombic model with different constraints

**Instrument**	**Scattering plane**	**Q**	I_{calc} **(cts per** 5 **min)**				I_{exp} **(cts per** 5 **min)**
			No constraints	One type of kagome	No interlayer canting	Net moment constraint	
HB-1A	(H K 0)	[0.5 0.5 0]	581	618	581	581	549(47)
		[1.5 1.5 0]	116	172	117	116	92(50)
	(H 0 L)	[0.5 0 1]	249	154	264	235	274(82)
		[1.5 0 2]	224	255	224	224	180(26)
		[0.5 0 2]	2	334	2	3	238(99)
		[0.5 0 3]	177	10	174	178	150(109)
SPINS	(H K 0)	[0.5 0.5 0]	926	985	926	926	816(51)
BT-7	(H K 0)	[0.5 0.5 0]	2090	2223	2090	2089	2207(334)
		[1 0 0]	24812	14624	21078	28927	23668(1512)
		[1 1 0]	5522	5836	6180	4532	5859(445)
	(H 0 L)	[0.5 0 1]	690	428	732	651	1095(209)
		[0.5 0 3]	693	41	682	700	577(115)
		[1 0 0]	33555	19777	28505	39119	35777(2191)
	(H H L)	[1 1 1]	3359	3386	3358	3359	3345(181)
		[1 1 0]	6078	6424	6803	4989	5892(338)
		[0.5 0.5 0]	2270	2414	2270	2269	2716(132)
		[0.5 0.5 2]	124	820	139	107	203(150)
		[0.5 0.5 1]	27	261	36	21	242(417)
		[0.5 0.5 3]	11	44	9	13	220(223)

TABLE 25: Contributions to the calculated intensities of magnetic Bragg peaks from different Cu^{2+} sites in the orthorhombic model

**Instrument**	**Scattering plane**	**Q**	**Orthorhombic model**		
			I_{calc} (cts per 5 min)		
			All	Only kagome	Only interlayer
HB-1A	(H K 0)	[0.5 0.5 0]	581	86	220
		[1.5 1.5 0]	116	25	34
	(H 0 L)	[0.5 0 1]	249	5	185
		[1.5 0 2]	224	39	76
		[0.5 0 2]	2	96	71
		[0.5 0 3]	177	2	160
SPINS	(H K 0)	[0.5 0.5 0]	926	137	351
BT-7	(H K 0)	[0.5 0.5 0]	2090	309	792
		[1 0 0]	24812	25814	55
		[1 1 0]	5522	7665	175
	(H 0 L)	[0.5 0 1]	690	14	514
		[0.5 0 3]	693	6	629
		[1 0 0]	33555	34910	75
	(H H L)	[1 1 1]	3359	3407	6
		[1 1 0]	6078	8437	193
		[0.5 0.5 0]	2270	335	860
		[0.5 0.5 2]	124	341	877
		[0.5 0.5 1]	27	30	0.6
		[0.5 0.5 3]	11	8	0.6

F. Including interlayer moments into calculations for barlowite 2

Based on the pinwheel *q=0* kagome model, we can estimate the FM exchange couplings between the kagome and interlayer Cu^{2+}s relative to the AFM couplings in the kagome plane. If we consider only NN couplings and assume that the coupling between the NN kagome spins is J ($J > 0$), then the total FM coupling between an interlayer spin and its six NN kagome spins is $-2\alpha J - 4\beta J$ when this interlayer site is fully occupied (Figure 24, inset). The average FM couplings for different interlayer sites are obtained by taking the site occupancies into account (see Tables 3 and 9).

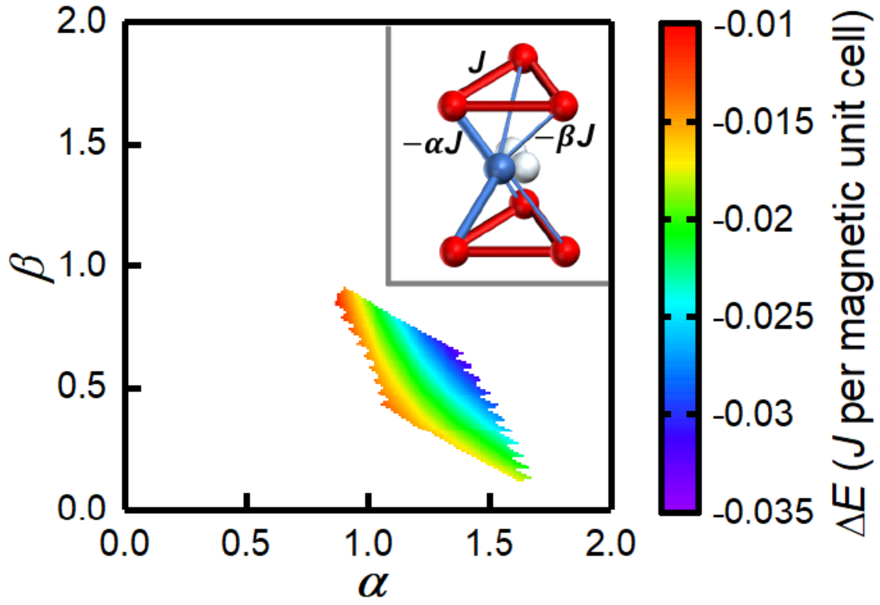

FIG. 24: Contour map showing the energy difference between the pinwheel and the perfect *q=0* kagome configurations ($\Delta E = E_{\text{pinwheel}} - E_{\text{perfect}}$), as described in the text. The inset shows the exchange couplings considered in this calculation. Red and blue/white spheres represent the kagome and interlayer Cu^{2+} ions, respectively. The couplings between the NN kagome spins are assumed to be the same (J, red lines). However, since the interlayer Cu^{2+} ion randomly occupies one of the three possible off-center interlayer sites (blue sphere) with some probability, there are two types of exchange paths between this site and its NN kagome spins. These couplings are denoted by $-\alpha J$ (thick blue lines) and $-\beta J$ (thin blue lines), respectively.

The classical-spin-based energy is investigated using the moment sizes from our pinwheel *q=0* kagome model (Table 19). The out-of-plane rotation angle of the kagome spins is fixed to the value from our model, but the in-plane rotation angles of both the kagome and interlayer spins are treated as tunable parameters. Because the energy only depends on the relative angles between the spins, we fix the in-plane rotation angle of the kagome spin $K1$. Therefore, for arbitrary α and β, the best in-plane rotation angles of the other spins can be determined by searching for the minimum energy configuration. This minimum energy is also compared with

the energy of the perfect *q=0* spin arrangement to determine which state is more energetically favorable. This calculation is done for a grid of α and β in the range of 0 to 2 with a step size of 0.01, and the energy difference between the pinwheel and the perfect *q=0* configuration is shown as a contour map in Figure 24. The colored region is where a) the pinwheel *q=0* structure has lower energy than the perfect *q=0* spin configuration, and b) the best in-plane rotation angles are close to the values from our neutron scattering refinement results (Table 19) with deviations no larger than 5° (approximately the error bars in the refinement). Within this colored region, the lowest energy state is achieved at $\alpha = 1.39$ and $\beta = 0.64$. Interestingly, the strong FM interactions obtained here match quite well with a previous report based on DFT calculations ($\alpha = 1.16$ and $\beta = 0.18$).[31] The average FM couplings for the three interlayer sites are $-0.96J$ (43% occupied), $-0.89J$ (33% occupied), and $-0.82J$ (24% occupied), respectively.

The robustness of the values of α and β against a change in moment sizes is also tested. When the kagome moment sizes are varied from 0.16 to 0.23 μ_B (the values of the kagome moments from the refinement), the best kagome in-plane rotation angle shows a deviation from the neutron scattering refinement result (Table 19) smaller than 17°.

We can attempt to understand the phase transitions upon cooling by modeling the effect of the interlayer spins $\vec{L}$ on the kagome spins $\vec{S}$ as an effective Zeeman field, i.e., $J_\mathrm{FM}\vec{L}\cdot\vec{S} \sim \vec{h}_\mathrm{eff}\cdot\vec{S}$, where $\vec{h}_\mathrm{eff} \sim J_\mathrm{FM}\langle\vec{L}\rangle$. From the DMRG calculations, the kagome plane forms a VBC ordered ground state with a finite spin gap, e.g., $\Delta \sim 0.15J$ for $J' = 0.95J$. Our experimental values for the interlayer moment (< 0.1 μ_B) in the intermediate temperature regime $T_\mathrm{N2} < T < T_\mathrm{N1}$ yield an effective Zeeman field $h_\mathrm{eff} < 0.04J$, which is too small to close the VBC spin gap, consistent with the kagome moments' absence of order. However, the interlayer ordered moment (and, therefore, h_eff) continues to increase upon cooling. As a result, it becomes strong enough to destabilize the pinwheel VBC state and drive the system into the pinwheel *q=0* state with long-range magnetic order.

VII. FIRST-ORDER PHASE TRANSITION BETWEEN THE PINWHEEL VBC AND QSL

We performed DMRG calculations of the ground state energy of the simplified model for barlowite **2** using the "hysteresis plot" method introduced in Ref. [5] to determine the nature of the phase transition. We find a first-order phase transition point between the pinwheel VBC phase and the QSL. The ground state energy is calculated along four paths of the Hamiltonian $H(J')$ for a 144-site 6-leg cylinder with two numbers of kept states, $m = 1600$ and 2000. A small step size $0.001J$ is taken in each of four adiabatic paths shown in Figure 25. Along the paths, the system shows a clear crossing at $J'_c = 0.998(1)$. This value is close to 1, but somewhat smaller, with a deviation larger than the resolution of our simulation.

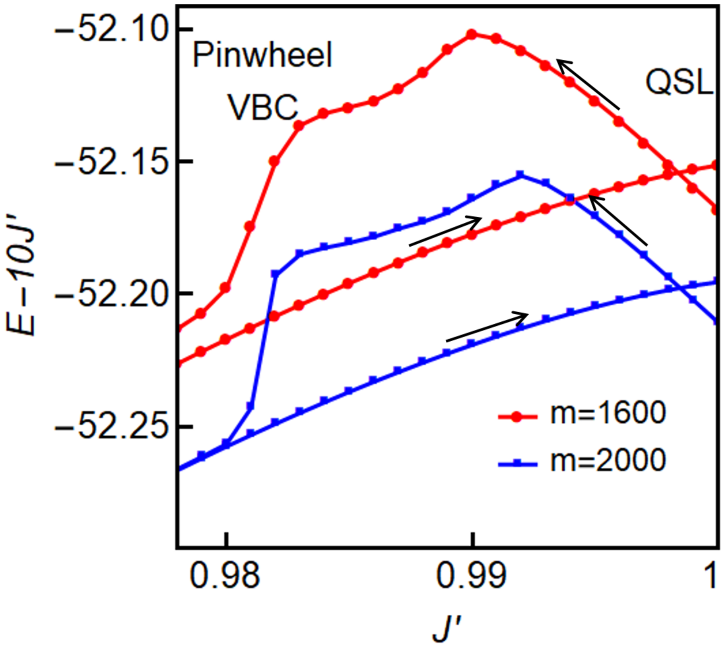

FIG. 25: Plot of the ground state energy of the simplified model for barlowite **2** for a 144-site 6-leg cylinder for two values of the kept states m. For each of the four paths, the Hamiltonian parameter J' is adiabatically tuned for every three DMRG sweeps to resemble the evolution of the system under changing J'. A linear subtraction of the energy E with J' has been applied.

[1] Sachdev, S. *Phys. Rev. B* **45,** 12377 (1992).
[2] Ran, Y., Hermele, M., Lee, P. A. & Wen, X.-G. *Phys. Rev. Lett.* **98,** 117205 (2007).
[3] Hermele, M., Ran, Y., Lee, P. A. & Wen, X.-G. *Phys. Rev. B* **77,** 224413 (2008).
[4] Jiang, H. C., Weng, Z. Y. & Sheng, D. N. *Phys. Rev. Lett.* **101,** 117203 (2008).
[5] Yan, S., Huse, D. A. & White, S. R. *Science* **332,** 1173–1176 (2011).
[6] Depenbrock, S., McCulloch, I. P. & Schollwöck, U. *Phys. Rev. Lett.* **109,** 067201 (2012).
[7] Jiang, H.-C., Wang, Z. & Balents, L. *Nat. Phys.* **8,** 902–905 (2012).
[8] Hao, Z. & Tchernyshyov, O. *Phys. Rev. B* **87,** 214404 (2013).
[9] He, Y.-C., Zaletel, M. P., Oshikawa, M. & Pollmann, F. *Phys. Rev. X* **7,** 031020 (2017).
[10] Balents, L. *Nature* **464,** 199–208 (2010).
[11] Norman, M. R. *Rev. Mod. Phys.* **88,** 041002 (2016).
[12] Mendels, P. & Bert, F. *C. R. Phys.* **17,** 455–470 (2016).
[13] Savary, L. & Balents, L. *Rep. Prog. Phys.* **80,** 016502 (2017).
[14] Braithwaite, R. S. W., Mereiter, K., Paar, W. H. & Clark, A. M. *Mineral. Mag.* **68,** 527–539 (2004).
[15] Shores, M. P., Nytko, E. A., Bartlett, B. M. & Nocera, D. G. *J. Am. Chem. Soc.* **127,** 13462–13463 (2005).
[16] Han, T.-H. *et al.* *Nature* **492,** 406–410 (2012).
[17] Fu, M., Imai, T., Han, T.-H. & Lee, Y. S. *Science* **350,** 655–658 (2015).
[18] Zhu, W., Gong, S. S. & Sheng, D. N. *Phys. Rev. B* **92,** 014424 (2015).
[19] Mei, J.-W., Chen, J.-Y., He, H. & Wen, X.-G. *Phys. Rev. B* **95,** 235107 (2017).
[20] Liao, H. J. *et al.* *Phys. Rev. Lett.* **118,** 137202 (2017).
[21] Hastings, M. B. *Phys. Rev. B* **63,** 014413 (2000).
[22] Syromyatnikov, A. V. & Maleyev, S. V. *Phys. Rev. B* **66,** 132408 (2002).
[23] Nikolic, P. & Senthil, T. *Phys. Rev. B* **68,** 214415 (2003).
[24] Singh, R. R. P. & Huse, D. A. *Phys. Rev. B* **76,** 180407(R) (2007).
[25] Kim, J.-H. *et al.* *Phys. Rev. Lett.* **101,** 107201 (2008).
[26] Yoshida, H. *et al.* *Nat. Commun.* **3,** 860 (2012).
[27] Freedman, D. E. *et al.* *J. Am. Chem. Soc.* **132,** 16185–16190 (2010).
[28] Han, T.-H. *et al.* *Phys. Rev. B* **94,** 060409(R) (2016).
[29] Elliott, P., Cooper, M. A. & Pring, A. *Mineral. Mag.* **78,** 1755–1762 (2014).
[30] Han, T.-H., Singleton, J. & Schlueter, J. A. *Phys. Rev. Lett.* **113,** 227203 (2014).
[31] Jeschke, H. O. *et al.* *Phys. Rev. B* **92,** 094417 (2015).
[32] Han, T.-H., Isaacs, E. D., Schlueter, J. A. & Singleton, J. *Phys. Rev. B* **93,** 214416 (2016).
[33] Smaha, R. W., He, W., Sheckelton, J. P., Wen, J. & Lee, Y. S. *J. Solid State Chem.* **268,** 123–129 (2018).
[34] Feng, Z. *et al.* *Phys. Rev. B* **98,** 155127 (2018).
[35] Tustain, K., Nilsen, G. J., Ritter, C., da Silva, I. & Clark, L. *Phys. Rev. Mater.* **2,** 111405(R) (2018).
[36] Liu, Z., Zou, X., Mei, J.-W. & Liu, F. *Phys. Rev. B* **92,** 220102(R) (2015).
[37] Guterding, D., Valentí, R. & Jeschke, H. O. *Phys. Rev. B* **94,** 125136 (2016).
[38] Feng, Z. *et al.* *Chinese Phys. Lett.* **34,** 077502 (2017).
[39] Wei, Y. *et al.* Preprint at `http://arxiv.org/abs/1710.02991` (2017).

[40] Pasco, C. M. *et al.* *Phys. Rev. Mater.* **2,** 044406 (2018).
[41] Henderson, A. *et al.* *Chem. Commun.* **55,** 11587–11590 (2019).
[42] Warren, B. *X-Ray Diffraction* (Dover Publications, 1990).
[43] Reisinger, S. A., Tang, C. C., Thompson, S. P., Morrison, F. D. & Lightfoot, P. *Chem. Mater.* **23,** 4234–4240 (2011).
[44] Kato, H., Kato, M., Yoshimura, K. & Kosuge, K. *J. Phys. Condens. Matter* **13,** 9311–9333 (2001).
[45] White, S. R. *Phys. Rev. Lett.* **69,** 2863–2866 (1992).
[46] Jeschke, H. O., Salvat-Pujol, F. & Valentí, R. *Phys. Rev. B* **88,** 075106 (2013).
[47] Han, T.-H., Chu, S. & Lee, Y. S. *Phys. Rev. Lett.* **108,** 157202 (2012).
[48] Wang, J., Zhou, Z., Zhang, W., Garoni, T. M. & Deng, Y. *Phys. Rev. E* **87,** 052107 (2013).
[49] Bert, F. *et al.* *Phys. Rev. B* **76,** 132411 (2007).
[50] Han, T. H. *et al.* *Phys. Rev. B* **83,** 100402(R) (2011).
[51] Helton, J. S. *et al.* *Phys. Rev. Lett.* **98,** 107204 (2007).
[52] Matan, K. *et al.* *Nat. Phys.* **6,** 865–869 (2010).
[53] Messio, L., Cépas, O. & Lhuillier, C. *Phys. Rev. B* **81,** 064428 (2010).
[54] Huh, Y., Fritz, L. & Sachdev, S. *Phys. Rev. B* **81,** 144432 (2010).
[55] Suttner, R., Platt, C., Reuther, J. & Thomale, R. *Phys. Rev. B* **89,** 020408(R) (2014).
[56] Fejer, E. E., Clark, A. M., Couper, A. G. & Elliott, C. J. *Mineral. Mag.* **41,** 433–436 (1977).
[57] Burns, P. C., Cooper, M. A. & Hawthorne, F. C. *Can. Mineral.* **33,** 633–639 (1995).
[58] Feng, Z. *et al.* *Chinese Phys. Lett.* **36,** 017502 (2019).
[59] Jambor, J. L., Dutrizac, J. E., Roberts, A. C., Grice, J. D. & Szymanski, J. T. *Can. Mineral.* **34,** 61–72 (1996).
[60] Malcherek, T., Mihailova, B. & Welch, M. D. *Phys. Chem. Miner.* **44,** 307–321 (2017).
[61] Wills, A. S. & Henry, J.-Y. *J. Phys. Condens. Matter* **20,** 472206 (2008).
[62] Clark, B. K., Kinder, J. M., Neuscamman, E., Chan, G. K.-L. & Lawler, M. J. *Phys. Rev. Lett.* **111,** 187205 (2013).
[63] Bruker AXS Inc. (Madison, 2016).
[64] Sheldrick, G. M. *Acta Crystallogr. C* **71,** 3–8 (2015).
[65] Dolomanov, O. V., Bourhis, L. J., Gildea, R. J., Howard, J. A. K. & Puschmann, H. *J. Appl. Crystallogr.* **42,** 339–341 (2009).
[66] Toby, B. H. & Von Dreele, R. B. *J. Appl. Crystallogr.* **46,** 544–549 (2013).
[67] Lynn, J. W. *et al.* *J. Res. NIST* **117,** 61–79 (2012).
[68] Abel, E. T. *et al.* *Phys. Rev. B* **76,** 214304 (2007).
[69] Shirane, G., Shapiro, S. M. & Tranquada, J. M. *Neutron Scattering with a Triple-Axis Spectrometer: Basic Techniques* (Cambridge University Press, 2002).
[70] Aroyo, M. I., Kirov, A., Capillas, C., Perez-Mato, J. M. & Wondratschek, H. *Acta Crystallogr. A* **62,** 115–128 (2006).
[71] Aroyo, M. I. *et al.* *Z. Krist.-Cryst. Mater.* **221,** 15–27 (2006).
[72] Aroyo, M. I. *et al.* *Bulg. Chem. Commun.* **43,** 183–197 (2011).
[73] Perez-Mato, J. M. *et al.* *Annu. Rev. Mater. Res.* **45,** 217–248 (2015).
[74] Momma, K. & Izumi, F. *J. Appl. Crystallogr.* **44,** 1272–1276 (2011).
